# The role of the renin-angiotensin system (RAS) in salinity adaptation in Pacific white shrimp (*Litopenaeus vannamei*)

**DOI:** 10.3389/fendo.2022.1089419

**Published:** 2022-12-15

**Authors:** Ardavan Farhadi, Yan Liu, Chang Xu, Xiaodan Wang, Erchao Li

**Affiliations:** ^1^ Key Laboratory of Tropical Hydrobiology and Biotechnology of Hainan Province, Hainan Aquaculture Breeding Engineering Research Center, College of Marine Sciences, Hainan University, Haikou, Hainan, China; ^2^ School of Life Sciences, East China Normal University, Shanghai, China

**Keywords:** adaptation, osmoregulation, renin-angiotensin system (RAS), shrimp, gene experession

## Abstract

The renin-angiotensin system (RAS) is a hormonal system that plays an important role in the regulation of blood pressure and cardiovascular homeostasis in mammals. In fishes, the RAS pathway participates in osmoregulation and salinity adaptation. However, the role of the RAS pathway in invertebrates, particularly in crustaceans, remains unknown. In this study, four key genes of the RAS pathway (*LV-ACE*, *LV-APN*, *LV-AT_1_R*, and *LV-RR*) were cloned, characterized, and their expression levels were detected in the eyestalk, hepatopancreas, and muscle of *Litopenaeus vannamei* during long-term and short-term low salinity stress. The results showed that *LV-ACE*, *LV-APN*, *LV-AT_1_R*, and *LV-RR* encode 666, 936, 175, and 323 amino acids, respectively. Low salinity stress downregulated the expression levels of *LV-ACE*, *LV-APN*, *LV-AT_1_R*, and *LV-RR* in *L. vannamei*, indicating that the RAS pathway was suppressed under low salinity. Moreover, these genes play important roles in the regulation of drinking rate, controlling urine output, blood glucose, and blood pressure, indicating that their downregulation probably affected the homeostasis of shrimps. These findings provide novel insights into the mechanism of salinity adaptation in *L. vannamei*.

## Introduction

**Figure 1:**
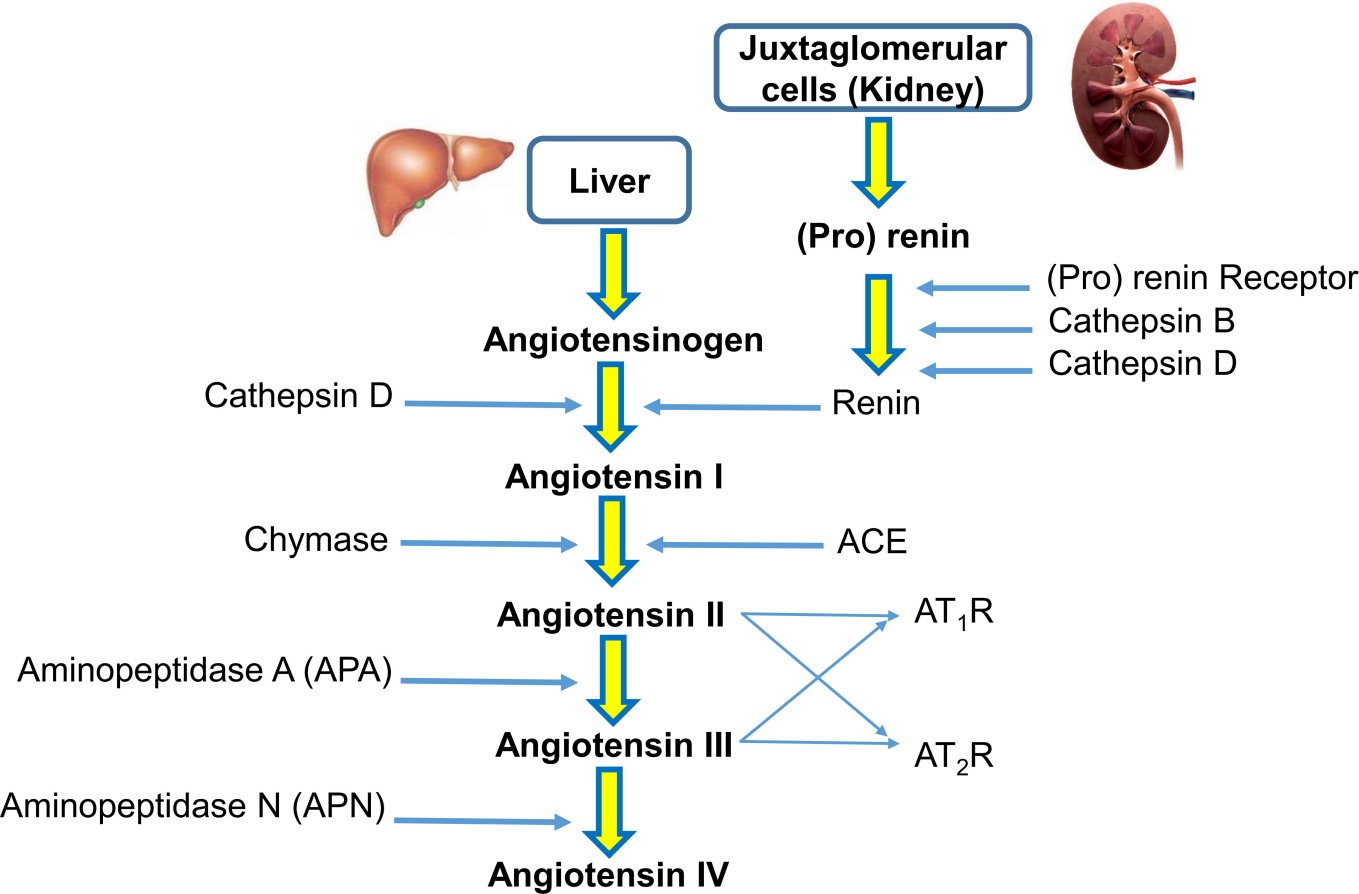
A simplified view of the renin-angiotensin system (RAS). In the RAS, (pro)renin activation is caused by its binding to the (pro)renin receptor. Cathepsins B and D are also renin-activating enzymes. Renin and cathepsin D converts angiotensinogen to angiotensin I, which is acted upon by angiotensin-converting enzyme (ACE) and chymase to produce angiotensin II. Aminopeptidase A (APA) converts angiotensin II to angiotensin III. Angiotensin II and angiotensin III act on the AT1R receptor and AT2R receptor. Aminopeptidase N (APN) converts angiotensin III to angiotensin IV.

The renin-angiotensin system (RAS) is a hormonal system that modulates blood pressure and cardiovascular homeostasis in vertebrates (1). A reduction in blood pressure induces the release of renin from the juxtaglomerular apparatus cells of the kidney into the cardiovascular system. In this process, renin cleaves the precursor protein angiotensinogen (AGT) into angiotensin I. Later, angiotensin-converting enzyme (ACE) cleaves angiotensin I at the carboxyl end to synthesize angiotensin II. Angiotensin II is known to be one of the main active peptides in this pathway. Angiotensin II affects the organs and tissues that modulate blood pressure by renal reabsorption of sodium and water and systemic vasoconstriction (2). To exert its biological functions, angiotensin II must bind to AT_1_R and AT_2_R receptors. It seems that the AT_1_R receptor regulates the main physiological functions of angiotensin II related to osmoregulation and fluid balance. In contrast, the AT_2_R receptor is associated with cellular remodeling processes, and stimulation of the AT_2_R receptor has suppressive effects on the cardiovascular system Aminopeptidase N (APN) converts angiotensin III to angiotensin IV (3). A simplified view of the RAS pathway is presented in **Figure 1**.

It is well known that the RAS system plays a key role in the regulation of salt and osmoregulation in mammals (4). Studies on fishes revealed that the osmoregulatory functions of angiotensin also play a crucial role in dealing with salinity challenges (5). In teleost fish, RAS participates in osmoregulation and salinity adaptation by regulating the drinking rate and blood pressure (6). Zebrafish larvae cultured under different salinities displayed changes in the expression levels of the *ren* and *angiotensin II* genes, indicating the important role of the RAS in salinity adaptation (7). Studies have shown that exposure to high salinity activates the RAS pathway in fishes. For example, the mRNA levels of hepatic *angiotensinogen* in rainbow trout were elevated in a hyperosmotic environment (5). In European eel and Japanese eel, the circulating levels of angiotensin II in plasma were higher in seawater than in freshwater (8, 9). A similar result was recorded for euryhaline bullshark, and the concentration of angiotensin II protein was significantly elevated during a long-term transfer from freshwater to seawater (10). Rapid changes in water salinity from saline water to freshwater resulted in a rapid decrease in plasma angiotensin in river lamprey (11). There is limited information regarding the role of the RAS pathway in crustaceans. In a recent study, the transcriptome analysis revealed that the RAS pathway was activated in Giant Freshwater Prawn (*Macrobrachium rosenbergii*) under hypotonic stress (12). Our previous study showed that the RAS pathway was suppressed under low salinity in the eyestalk of the Pacific white shrimp *Litopenaeus vannamei* (13).

The Pacific white shrimp (*L. vannamei*) is considered an economically important species, and its culture is increasing to meet the high demands of the market (14). This species is a euryhaline species due to its high capability to tolerate a wide range of salinities from 0.5 to 40 ppt (15). Salinity is an abiotic factor that affects the growth, metabolism, immunity, and survival of shrimp. *L. vannamei* is a euryhaline species that can tolerate a wide range of salinities from 0.5 to 40 ppt (15). However, *L. vannamei* has low growth, high mortality, and low disease resistance when cultured under low salinity conditions (16). Salinity adaptation is a complex process that involves several biological pathways and different organs. For example, the hepatopancreas is known to provide the required energy for gills, and gills maintain the ionic balance (17). Various adaptive mechanisms must be triggered in *L. vannamei* to tolerate a wide range of salinity changes. In our previous study, transcriptome analysis revealed that the RAS pathway and its related genes might play important roles in salinity adaptation in *L. vannamei*, but the detailed mechanisms remain unknown (13).

Previous studies have provided useful information regarding the role of the RAS pathway in the regulation of blood pressure and cardiovascular homeostasis in vertebrates. However, there is limited information regarding the role of the RAS pathway in invertebrates. Unlike vertebrates, *L. vannamei* has an open circulatory system and different sodium concentrations in the blood; therefore, the RAS might have different regulatory mechanisms and roles in *L. vannamei* than in vertebrates. In the open circulatory system, the blood (hemolymph) pressure in the body is much lower than in the closed circulatory system, and there is no true heart or capillaries (instead, there are open sinuses, and the movements of body muscles help the hemolymph flow throughout the sinuses). Moreover, the concentration of sodium in the hemolymph of *L. vannamei* is about 299 mM, while the sodium concentration in the human blood is about 136-145 mM (18).

The present study was carried out to expand the knowledge about the RAS pathway and its related genes in *L. vannamei* during low salinity stress. This study aims to 1) identify and characterize key genes in the RAS pathway, such as *LV-ACE* (angiotensin-converting enzyme), *LV-APN* (aminopeptidase N), *LV-AT_1_R* (angiotensin I receptor), and *LV-RR* (renin receptor), and 2) investigate the expression levels of *LV-ACE*, *LV-APN*, *LV-AT_1_R*, and *LV-RR* in different tissues under long-term and short-term low salinity stress. The results of this study help to understand how the RAS pathway and its related genes participate in the osmoregulation of *L. vannamei*.

## Materials and methods

### Experimental animals and sampling

Juvenile shrimp (mean weight 1.80 ± 0.16 g) were purchased from a shrimp farm in Shenzhen, China. Shrimp were adapted to the experimental conditions for 1 week (at a salinity of 20). During a one-week period, the salinity of the aquariums was adjusted to 25 ppt and 3 ppt by adding seawater and freshwater, respectively. Shrimp were divided into two salinity groups: 25 ppt (control group) and 3 ppt (low salinity group). For long-term low salinity stress, the experiment was conducted for 8 weeks. For short-term low salinity stress, the samples were collected at 0 h, 3 h, 6 h, 12 h, 24 h, 48 h, 72 h, and 96 h. Shrimp were fed twice daily (at 08:00 and 16:00) with commercial pelleted feed (46% crude protein, 8% crude lipid, 36% carbohydrates, 10% moisture, 11% ash, and 16.7 kJ/g digestible energy). The water temperature, photoperiod, pH, dissolved oxygen, and ammonia nitrogen ranged from 26-28°C, 2 light:12 dark, 7.4-7.8, 4.7-6.5 mg/L, and < 0.02 mg/L, respectively. After dissection, eyestalk, hepatopancreas, and muscle samples were stored in RNAKeeper Tissue Stabilizer (Vazyme, Nanjing) and incubated for 24 h at 4°C. Later, the samples were transferred to -80°C until RNA extraction. The experiment was conducted under the principles of the Guide of the Institutional Animal Care and Use Ethics Committee of East China Normal University.

### Gene cloning

The fragments of *LV-ACE*, *LV-APN*, *LV-AT_1_R*, and *LV-RR* genes were screened and identified from our transcriptome data (Accession number: SRP048814) by using blast comparison (13). Total RNA was extracted using TRIzol reagent (Invitrogen, Shanghai, China) following the manufacturer’s instructions. The quality and concentration of the extracted RNA were tested using an Agilent 2100 Bioanalyzer (Agilent Technologies, USA) and a Nanodrop 2000 spectrophotometer (ND-2000, Gene Company Limited), respectively. First-strand cDNA was synthesized using FastKing gDNA Dispelling RT SuperMix (TIANGEN, China). PCR amplification was carried out in a total volume of 25 µl, including 12.5 μL 2X Taq PCR master mix, 4 µl cDNA, 0.4 µl forward and reverse primers, and 7.7 μL RNase Free dH_2_O. The reaction procedure was set at 94 °C for 3 min as preincubation, PCR amplification was performed for 34 cycles (94 °C for 30 s; 55 °C for 30 s; 72 °C for 1 min), and 5 min of extension at 72°C. The amplified products were sent to a biological company (Meiji Biotech Co., Ltd., Shanghai, China) for sequencing, and then the obtained sequences were spliced to obtain the final sequence. The primers were designed using Primer Premier Software 5.0 (PREMIER Biosoft International, USA). All primers used are presented in **Table 1**.

**Table 1:** Primers used for cloning and qRT−PCR of *LV-ACE*, *LV-APN*, *LV-AT_1_R*, and *LV-RR*.

Primer name	Sequences (5′-3′)	Description	Tm (°C)	Product size
LV-ACE	Forward: CGCCTCCTGGAACTACGATTCC	For cloning	59.5	2,326 Bp
	Reverse: TGAACCACGAAGCTGACGAAGT	For cloning	59.2	
LV-APN	Forward: TTGTTCTGCTCGCCCTCGGT	For cloning	55.9	3,100 Bp
	Reverse: GCCACTGCTCCTGTTCATAGCC	For cloning	60.3	
LV-AT_1_R	Forward: TGTGGATGCCTCACCGACAGTT	For cloning	60.8	593 Bp
	Reverse: GCCGCACTAGCAGGTTGATGAC	For cloning	62.0	
LV-RR	Forward: GCGTGCAACCCACCTTGATGAT	For cloning	60.7	1,116 Bp
	Reverse: ACGTCCAGGATCCATGGTAGCC	For cloning	60.5	
LV-ACE-qRT	Forward: CGTGAGTCTGGAGGAGGAA	For qRT-PCR	55.3	
	Reverse: GGTGATGTCGGAATCGTAGTT	For qRT-PCR	54.8	
LV-APN-qRT	Forward: TCGTGAAGGTGACAAGGAG	For qRT-PCR	54.0	
	Reverse: CGTGGTGCTGGAAGAGTT	For qRT-PCR	54.7	
LV-AT_1_R-qRT	Forward: TGCACCACCGTGAGACCGAA	For qRT-PCR	61.2	
	Reverse: GCCGCACTAGCAGGTTGATGAC	For qRT-PCR	60.8	
LV-RR-qRT	Forward: GGTCCACCCTTTCATCCGAGGT	For qRT-PCR	60.8	
	Reverse: GCAGCATCCACAAGAGCATCCA	For qRT-PCR	60.2	
*β-actin*	Forward: CCACGAGACCACCTACAAC	For qRT-PCR	54.8	
	Reverse: AGCGAGGGCAGTGATTTC	For qRT-PCR	54.8	

### Bioinformatic analysis

The protein sequences of *LV-ACE*, *LV-APN*, *LV-AT_1_R*, and *LV-RR* were compared by the BLAST search tool (available at https://blast.ncbi.nlm.nih.gov/Blast.cgi). The Compute pI/Mw tool was used to predict the isoelectric point and molecular weight of the proteins (available at https://web.expasy.org/compute_pi/). The three-dimensional (3D) protein structure and functional domains were predicted using SWISS-MODEL (https://swissmodel.expasy.org/interactive) and the Simple Modular Architecture Research Tool (https://smart.embl.de/), respectively. Signal peptide sites were predicted using the SignalP 5.0 tool (19). The secondary structure of proteins was predicted using the self-optimized prediction method with alignment (SOPMA) server. Mega X software (20) (bootstrap analysis of 1,000 replicates) was used to construct the phylogenetic tree by the neighbor-joining method. Multiple sequence alignment of proteins was performed using BioEdit software version 7.2 (http://bioedit.software.informer.com/7.2).

### Quantitative real-time PCR (qRT−PCR)

The expression patterns of *LV-ACE*, *LV-APN*, *LV-AT_1_R*, and *LV-RR* in different tissues (eyestalk, hepatopancreas, and muscle) under different salinity conditions were measured by *qRT−PCR* using the SYBR Green method (TIANGEN Biotech, Beijing, China). *qRT−PCR* was carried out in a total reaction volume of 20 µl on a 96-well PCR plate (Eppendorf, Germany) with three biological replicates for each sample. *qRT−PCR* was performed on a Bio-Rad CFX384 real-time PCR system (Bio-Rad, California, United States). The target genes and the housekeeping gene (*β-actin*) were amplified using gene-specific primers (**Table 1**). Gene expression data were analyzed using the 2^−ΔΔCT^ method (21).

### Bacterially expressed *LV-ACE* protein and preparation of antibody

A fragment sequence of *LV-ACE* was amplified using a PCR machine and then ligated with the prokaryotic expression plasmids pET28a (with the expected molecular weight of 21 kD) and pET32a (with the expected molecular weight of 35 kD). Later, pET28a was selected for further analysis. The recombinant protein was expressed in *Escherichia coli* BL21 (DE3). After an overnight incubation at 37°C, the bacteria were transferred into new media at a ratio of 1:100. The cultures were grown to mid-log phase at 37°C until the OD_600_ reached 0.6. The cultures were induced with isoprophyl-b-d-thiogalactopyranoside (IPTG) and purified by Ni^2+^-NTA affinity chromatography. The bacteria were sonicated using ultrasonic waves and then centrifuged at 1000 × g for 5 min. SDS−PAGE was used to analyze the collected sediment and supernatant. Ni-NTA spin columns were used to purify the protein, and the purified protein was detected using SDS−PAGE. New Zealand white rabbits were immunized with purified protein. The titers of antisera were assayed by enzyme-linked immunosorbent assay (ELISA). The antibodies were provided by a biological company (Youke Biotechnology Co., Ltd., Shanghai, China).

### Western blot

Total protein was extracted from the hepatopancreas of *L. vannamei* cultured under long-term low salinity and normal salinity with RIPA lysis buffer and quantified by the BCA method. After the separation of proteins by SDS−PAGE, proteins were transferred from the gel to a PVDF membrane. The membrane was blocked with blocking buffer (1% casein) for 2 h at room temperature. The first (rabbit anti-LV-ACE) and second antibodies (GAPDH) were diluted with PBST and incubated with the PVDF membranes. The PVDF membranes were exposed to X-ray film in a darkroom. After photographing the blots, the images were analyzed using ImageJ software.

### Statistical analysis

The experimental data were analyzed using SPSS version 21.0 (Chicago, Ill., USA). The normality and homoscedasticity of the data were verified using the Kolmogorov−Smirnov and Levene’s tests, respectively. Independent samples t tests were employed to determine the differences in the expression profiles of *LV-ACE*, *LV-APN*, *LV-AT_1_R*, and *LV-RR* under different salinity conditions. The significance level for all analyses was set at *P* < 0.05. In the present study, the experimental data are presented as the mean ± SD.

## Results

### Identification and characterization of *LV-ACE*, *LV-APN*, *LV-AT_1_R*, and *LV-RR*

The molecular masses of the LV-ACE, LV-APN, LV-AT_1_R, and LV-RR proteins were estimated to be 76.446, 108.381, 19.183, and 35.749 kDa, respectively. The estimated isoelectric points (IPs) for the LV-ACE, LV-APN, LV-AT_1_R, and LV-RR proteins were 5.78, 6.8, 4.73, and 4.95, respectively. The LV-ACE, LV-APN, LV-AT_1_R, and LV-RR proteins consist of 666, 936, 175, and 323 amino acid residues, respectively. The signal peptide sites and the secondary structure of the proteins are provided in **Supplementary Figures 1–4**.

### Phylogenetic analysis

**Figure 2:**
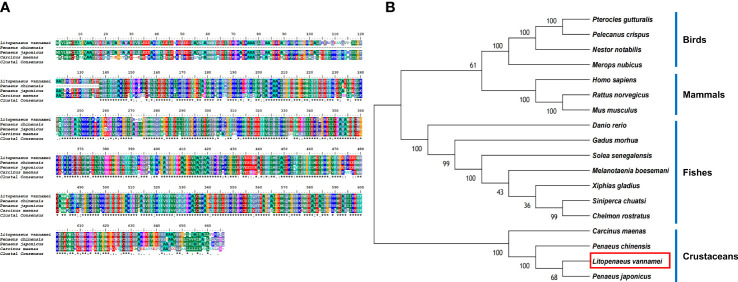
**(A)** Multiple alignments of deduced amino acids of LV-ACE protein and its homologs. Species names are listed on the left side. **(B)** The phylogenetic tree shows relationships between the ACE protein of *L. vannamei* (red rectangle) and those of other organisms. Species names are listed to the right of the tree.

The protein sequences of LV-ACE, LV-APN, LV-AT_1_R, LV-RR, and other proteins that were used for the bioinformatic analysis are provided as **Supplementary Files**. Bioinformatics analysis of the protein sequence of LV-ACE indicated its structural similarity to the angiotensin-converting enzyme-like family. BLAST analysis revealed that the LV-ACE protein shared the highest identity with ACE proteins in crustaceans such as *Penaeus chinensis* (94.83%), *Penaeus japonicus* (92.19%), and *Carcinus maenas* (72.06%), but the similarity of LV-ACE with other animal groups was less than 70% (**Figure 2A**). The constructed phylogenetic tree using 18 homologous proteins is shown in **Figure 2B**. In this tree, the homologous proteins are divided into four separate clades: crustaceans, fishes, mammals, and birds. The phylogenetic tree showed that the ACEs in crustaceans clustered together, indicating that they had a close genetic distance.

**Figure 3:**
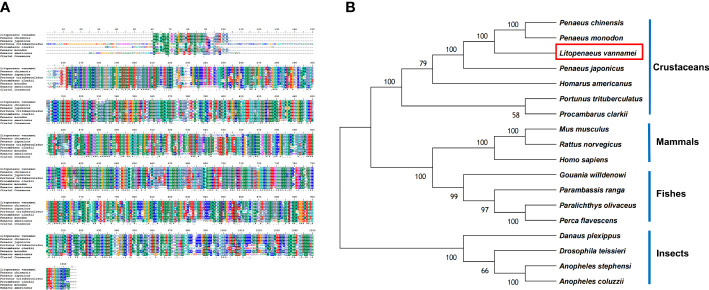
**(A)** Multiple alignments of deduced amino acids of LV-APN protein and its homologs. Species names are listed on the left side. **(B)** The phylogenetic tree shows relationships between the APN protein of *L. vannamei* (red rectangle) and those of other organisms. Species names are listed to the right of the tree.

Based on the bioinformatics analysis, LV-APN had a high structural similarity to aminopeptidase N-like proteins (**Figure 3A**). The LV-APN protein had the highest identity with aminopeptidase N-like proteins in crustaceans, including *Penaeus monodon* (82.46%), *P. chinensis* (81.41%), and *P. japonicus* (72.71%). The phylogenetic tree was constructed using 18 homologous proteins from different animal groups, including crustaceans, fishes, mammals, and insects (**Figure 3B**).

**Figure 4:**
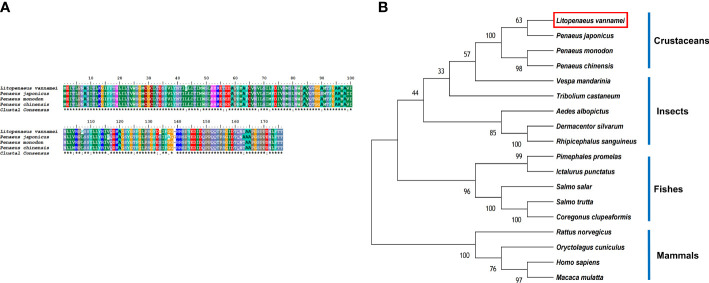
**(A)** Multiple alignments of deduced amino acids of LV-AT_1_R protein and its homologs. Species names are listed on the left side. **(B)** The phylogenetic tree shows relationships between the AT_1_R protein of *L. vannamei* (red rectangle) and those of other organisms. Species names are listed to the right of the tree.

The LV-AT_1_R protein had the highest structural similarity with type-1 angiotensin II receptor-associated protein-like in crustaceans (**Figure 4A**). BLAST analysis showed that the LV-AT_1_R protein shared the highest identity with *P. monodon* (93.71%), *P. chinensis* (93.14%), and *P. japonicus* (92.61%). The constructed phylogenetic tree using 18 homologous proteins from various species is presented in **Figure 4B**.

**Figure 5:**
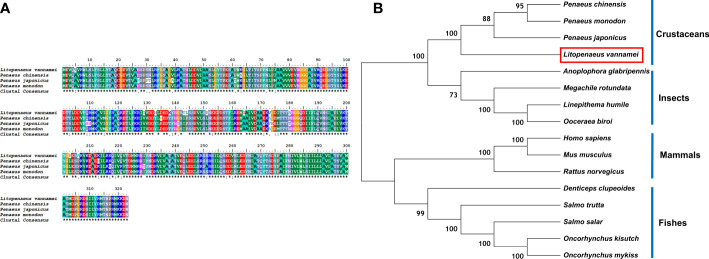
**(A)** Multiple alignments of deduced amino acids of LV-RR protein and its homologs. Species names are listed on the left side. **(B)** The phylogenetic tree shows relationships between the RR proteins of *L. vannamei* (red rectangle) and those of other organisms. Species names are listed to the right of the tree.

The comparison of the protein sequences of LV-RR in the NCBI database showed that this protein belongs to the renin receptor protein family (**Figure 5A**). The LV-RR protein had the highest identity with renin receptors in *P. monodon* (94.74%), *P. chinensis* (93.50%), and *P. japonicus* (88.85%). The phylogenetic tree for the LV-RR protein is shown in **Figure 5B**.

### Long-term low salinity stress: Expression profile of *LV-ACE*, *LV-APN*, *LV-AT_1_R*, and *LV-RR* in different tissues under different salinity conditions

**Figure 6:**
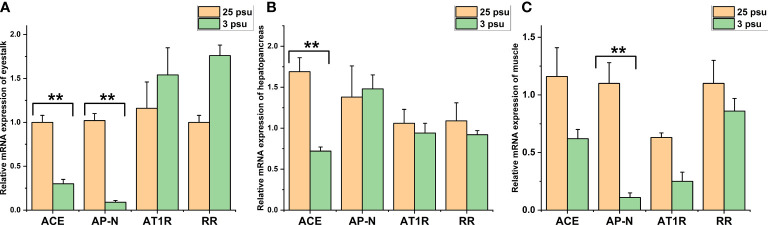
The expression levels of *LV-ACE*, *LV-APN*, *LV-AT_1_R*, and *LV-RR* in the eyestalk **(A)**, hepatopancreas **(B)**, and muscle **(C)** under long-term low salinity stress. Significant differences are indicated with ** marks (*P* < 0.05). Values are the mean ( ± SD). (N = 6).

The expression levels of *LV-ACE*, *LV-APN*, *LV-AT_1_R*, and *LV-RR* in the eyestalk, hepatopancreas, and muscle are presented in **Figure 6**. All four genes were expressed in the tested tissues. In the eyestalk, a downward trend was observed in the expression levels of *LV-ACE* and *LV-APN* under low salinity stress (*P* < 0.05). Low salinity stress did not affect the expression level of *LV-AT_1_R* or *LV-RR* in the eyestalk. In the hepatopancreas, the *LV-ACE* expression level was downregulated in the low salinity group compared to the control group (*P* < 0.05). The transcript levels of *LV-APN*, *LV-AT_1_R*, and *LV-RR* in the hepatopancreas were not affected by salinity changes (*P* > 0.05). In the muscle, the expression level of *LV-APN* was downregulated after long-term low salinity stress (*P* < 0.05). In addition, low salinity stress slightly decreased the expression levels of *LV-ACE, LV-AT_1_R*, and *LV-RR* in the muscle; however, no significant difference was found (*P* > 0.05).

### Short-term low salinity stress: Expression profile of *LV-ACE*, *LV-APN*, *LV-AT_1_R*, and *LV-RR* in different tissues under different salinity conditions

**Figure 7:**
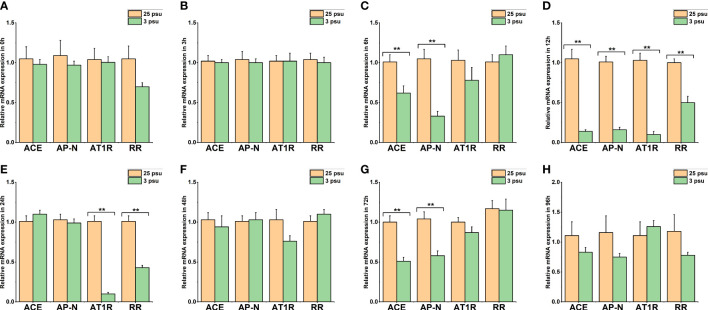
The expression levels of *LV-ACE*, *LV-APN*, *LV-AT_1_R*, and *LV-RR* in the eyestalk at 0 h **(A)**, 3 h **(B)**, 6 h **(C)**, 12 h **(D)**, 24 h **(E)**, 48 h **(F)**, 72 h **(G)**, and 96 h **(H)** of short-term low salinity stress. Significant differences are indicated with ** marks (*P* < 0.05). Values are the mean ( ± SD). (N = 6).

**Figure 8:**
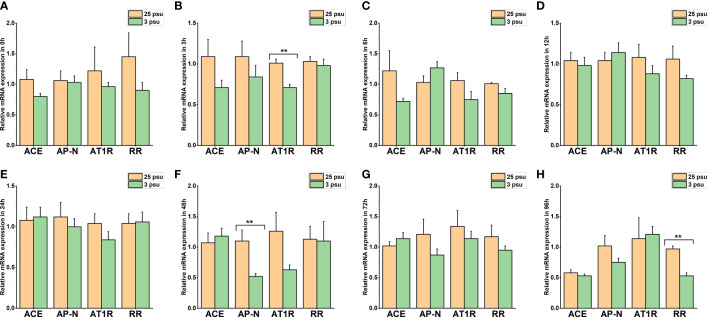
The expression levels of *LV-ACE*, *LV-APN*, *LV-AT_1_R*, and *LV-RR* in the hepatopancreas at 0 h **(A)**, 3 h **(B)**, 6 h **(C)**, 12 h **(D)**, 24 h **(E)**, 48 h **(F)**, 72 h **(G)**, and 96 h **(H)** of short-term low salinity stress. Significant differences are indicated with ** marks (*P* < 0.05). Values are the mean ( ± SD). (N = 6).

**Figure 9:**
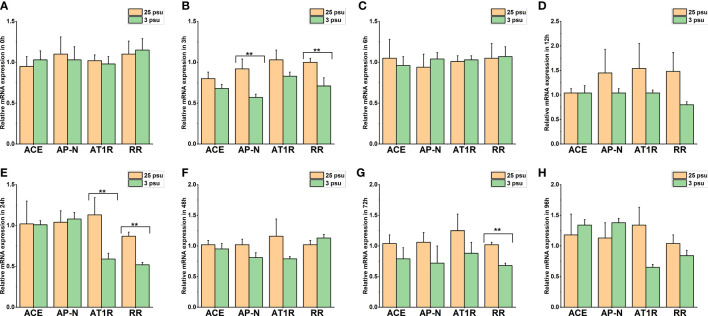
The expression levels of *LV-ACE*, *LV-APN*, *LV-AT_1_R*, and *LV-RR* in the muscle at 0 h **(A)**, 3 h **(B)**, 6 h **(C)**, 12 h **(D)**, 24 h **(E)**, 48 h **(F)**, 72 h **(G)**, and 96 h **(H)** of short-term low salinity stress. Significant differences are indicated with ** marks (*P* < 0.05). Values are the mean ( ± SD). (N = 6).

The expression profiles of *LV-ACE*, *LV-APN*, *LV-AT_1_R*, and *LV-RR* in the eyestalk, hepatopancreas, and muscle under short-term low salinity stress are presented in **Figures 7**–**9**, respectively.

**Figure 7** shows the mRNA expression of *LV-ACE*, *LV-APN*, *LV-AT_1_R*, and *LV-RR* in the eyestalk at 0 h, 3 h, 6 h, 12 h, 24 h, 48 h, 72 h, and 96 h of low salinity stress. The expression levels of the *LV-ACE* and *LV-APN* genes were significantly decreased at 6, 12, and 72 h (*P* < 0.05; **Figures 7C, D, G**). For the *LV-AT_1_R* and *LV-RR* genes, a significant downregulation was observed from 12 to 24 h in the low salinity group (3 ppt) compared to the 25 ppt salinity group (**Figures 7D, E**).

The mRNA expression profile of *LV-ACE*, *LV-APN*, *LV-AT_1_R*, and *LV-RR* in the hepatopancreas at 0 h, 3 h, 6 h, 12 h, 24 h, 48 h, 72 h, and 96 h is presented in **Figure 8**. The expression level of *LV-ACE* was not affected by short-term low salinity stress (*P* > 0.05). Based on the results (**Figure 8F**), the expression level of *LV-APN* in the hepatopancreas at 48 h decreased compared to that in the control group (*P* < 0.05). Downregulation of the expression of *LV-AT_1_R* and *LV-RR* was recorded at 3 h (**Figure 8B**) and 96 h (**Figure 8H**), respectively (*P* < 0.05).

**Figure 9** presents the expression profile of *LV-ACE*, *LV-APN*, *LV-AT_1_R*, and *LV-RR* in the muscle at 0 h, 3 h, 6 h, 12 h, 24 h, 48 h, 72 h, and 96 h under different salinity conditions. The mRNA expression of *LV-ACE* in the muscle remained intact during short-term low salinity stress (*P* > 0.05). Downregulation of the expression level of *LV-APN was observed* at 3 h (**Figure 9B**; *P* < 0.05). The expression of the *LV-AT_1_R* gene in the muscle significantly decreased at 24 h in comparison with the control group (**Figure 9E**; *P* < 0.05). For the *LV-RR* gene, the downregulation was recorded at 3, 24, and 72 h (**Figures 9B, E, G**; *P* < 0.05).

### Purification and expression of LV-ACE protein

**Figure 10:**
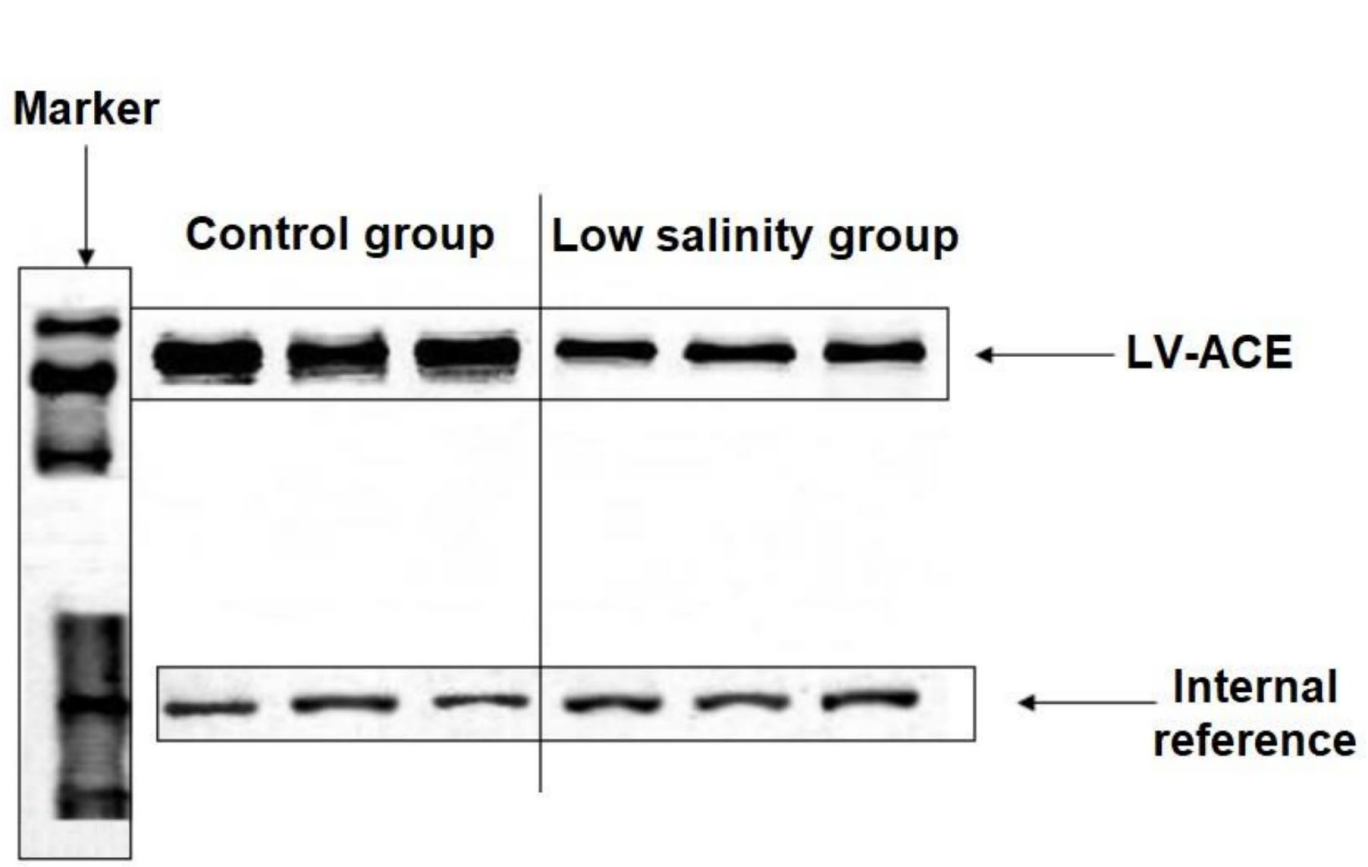
Western blot results of the expression of LV-ACE protein in the hepatopancreas under long-term low salinity and the control group. GAPDH was used as an internal reference.

The ligated protein fragments with prokaryotic expression plasmids pET28a and pET32a are shown in **Supplementary Figure 5A**. LV-ACE protein was successfully expressed after induction. The solubility of LV-ACE recombinant protein was determined by ultrasonic disruption of bacteria (**Supplementary Figure 5B**). After ultrasonic disruption, only LV-ACE recombinant protein appeared on SDS−PAGE, suggesting the presence of LV-ACE recombinant protein in the form of inclusion bodies. The SDS−PAGE for purification and quantification of LV-ACE recombinant protein is presented in **Supplementary Figures 5C, D**, respectively. Western blot results showed the expression of LV-ACE protein in the hepatopancreas under long-term low salinity and normal salinity conditions (**Figure 10**). The expression of LV-ACE protein in the hepatopancreas was decreased under long-term low salinity stress compared with the control group. The Western blot results were consistent with the qRT−PCR results.

## Discussion

It is well known that the RAS pathway is a key regulator of blood pressure and osmoregulation in mammals (1). However, for a better understanding of the role, evolution, and regulatory mechanism of the RAS pathway, it is pivotal to study the molecular characterization and expression analysis of RAS-related genes in other animal groups, such as invertebrates.

### Identification, characterization, and expression analysis of *LV-ACE*

Based on the sequence homology and phylogenetic analysis, the LV-ACE protein had the closest evolutionary relationship with ACE proteins in crustaceans. The phylogenetic tree revealed that ACE protein in crustaceans was distinct from other identified proteins in vertebrates and therefore might have a divergent evolutionary trend. Notably, the LV-ACE protein (666 aa) and ACE proteins in crustaceans (ranging from 529-666 aa) were much smaller than most of the ACE proteins in vertebrates (ranging from 1147-1324 aa).

*LV-ACE* was expressed in all the examined tissues in both the control and low salinity groups, indicating that *LV-ACE* might have a wide range of functions. Several studies have reported changes in the expression level of the *ACE* gene under salinity challenges in aquatic organisms (22, 23). In the present study, the expression analysis showed that *LV-ACE* in the eyestalk and hepatopancreas was downregulated after eight weeks of long-term low salinity stress. During the short-term low salinity stress, the downregulation in the mRNA level of *LV-ACE* was detected only in eyestalk but not in other tissues. In the eyestalk, the expression of *LV-ACE* decreased at 6 h, 12 h, and 72 h of short-term low salinity stress. A similar result was observed in our previous study, mRNA sequencing showed downregulation in the expression level of *LV-ACE* in the eyestalk of *L. vannamei* after long-term low salinity stress (13). The downregulation of *LV-ACE* in the eyestalk (but not in other organs) under short-term salinity stress could be related to the quick response of the eyestalk as an important regulatory organ for facing stressful situations. Eyestalk regulates energy metabolism by secreting crustacean hyperglycemic hormones (CHHs). The CHH neurohormones are involved in the stress responses by regulating the glucose level in the hemolymph (24). In mammals, salt intake regulates both plasma and tissue *ACE* expression (25). In rats, the mRNA expression level of *ACE* significantly increased after feeding with a diet containing a high level of salt compared to the control diet (25). The downregulation of *LV-ACE* under low saintly could be a response to low salt concentration in this group. In contrast to our findings, proteomics analysis exhibited an upregulation in the ACE protein under the low salinity group in the hepatopancreas of mud crab *Scylla paramamosain* (26). The ACE enzyme was initially isolated as a ‘hypertensin-converting enzyme’ in 1956 (27). This enzyme is present in several tissues and biological fluids. In humans, two isoforms of ACE have been identified: somatic ACE (sACE) and germinal ACE (gACE). Somatic ACE is present in different types of endothelial and epithelial cells (28). Germinal ACE is found particularly in germinal cells of male testis. In the RAS pathway, angiotensin I-(1-10) (Asp-Arg-Val-Tyr-Ile-His-Pro-PheHis-Leu) is cleaved into the octapeptide angiotensin II-(1-8) by ACE enzyme by removing the C-terminal dipeptide His-Leu (29). The catalytic mechanism of ACE in invertebrates is not clear. In humans, ACE activity depends on chloride ion concentration. Chloride mainly induces the active part of ACE and increases the binding of substrates (28). Each active domain of ACE exhibits different levels of sensitivity to different chloride concentrations (30). The C domain of sACE activates in the presence of chloride ions and is inactive in its absence. In contrast, the N domain is completely active at a very low chloride concentration and can even remain active in the absence of chloride (31). Seawater (salinity of 35 ppt) contains a chloride ion concentration of approximately 19,400 mg/L. The concentration of chloride ions in brackish water (salinity of 1-10 ppt) ranges from 500 to 5,000 mg/L. Freshwater (salinity of <0.5 ppt) has a lower chloride concentration, which can range from 1 to 250 mg/L (32). The concentration of chloride in the hemolymph of crustaceans is directly affected by the presence of this ion in the surrounding water (33). Therefore, the changes in ACE activity in the present study and other studies under different salinities might be related to the differences in the chloride ion concentration at different salinities.

### Identification, characterization, and expression analysis of *LV-APN*

The LV-APN protein had the highest similarity to aminopeptidase N-like proteins in *P. monodon* (82.46%), *P. chinensis* (81.41%), and *P. japonicus* (72.71%). The comparison and phylogenic analysis of aminopeptidase N-like proteins in 18 species from different animal groups (i.e., crustaceans, fishes, mammals, and insects) revealed that aminopeptidase N-like proteins in crustaceans clustered together and had a close genetic distance. The APN protein in all 18 species was almost the same size (ranging from 933-1065 aa).

The expression analysis of *LV-APN* showed that this gene was expressed in all the examined tissues (i.e., eyestalk, hepatopancreas, and muscle) in both the control and low salinity groups, suggesting that *LV-APN* probably has a wide functional range. In the present study, the expression level of *LV-APN* in the eyestalk and muscle was downregulated after eight weeks of long-term low salinity stress. Short-term low salinity stress significantly downregulated the expression level of *LV-APN* in the eyestalk, hepatopancreas, and muscle. In the eyestalk, the expression of *LV-APN* significantly decreased at 6 h, 12 h, and 72 h of short-term low salinity stress. For the hepatopancreas and muscle, the downregulation was observed at 48 h and 3 h of short-term low salinity stress, respectively. Similarly, mRNA sequencing of mud crab *S. paramamosain* megalopa showed that the expression level of *APN* was downregulated under low salinity (34). The activity of APN in crustaceans is not limited to salinity adaptation. APN activity in the hepatopancreas of the euryhaline crab (*Cyrtograpsus angulatus*) was changed in response to salinity, pH, and water temperature challenges (35). This enzyme plays an important role in the final stages of protein digestion in mammals, and its activity in the intestine is considered an important indicator of protein digestive capacity (36). Therefore, the increase in the activity of APN in the hepatopancreas can lead to an increase in amino acid availability, which can be used in biochemical adjustments during salinity stress in crustaceans (37). Considering the findings of the present study, previous studies, and the important role of APN in the RAS pathway, it seems that APN participates in salinity adaptation not only by affecting the enzymatic activity of the hepatopancreas and energy availability. The changes in genes related to the RAS pathway (i.e., *LV-ACE*, *LV-APN*, *LV-AT_1_R*, and *LV-RR*) in different tissues (i.e., eyestalk, hepatopancreas, and muscle) indicate that APN might also be involved in salinity adaptation through the RAS pathway.

### Identification, characterization, and expression analysis of *LV-AT_1_R*

Sequence homology and phylogenetic analysis revealed that *LV-AT_1_R* belongs to the type-1 angiotensin II receptor family. The structure and sequences of the AT_1_R protein in 18 various species were compared. Interestingly, AT_1_R proteins in crustaceans (175-176 aa), fishes (146-201 aa), and insects (151-173 aa) were smaller than AT_1_R proteins in mammals (358 aa). Moreover, the molecular mass and estimated IP of the AT_1_R protein in crustaceans (molecular mass: 19 kDa; IP: 4.7), fishes (molecular mass: 16-22 kDa; IP: 5-6), and insects (molecular mass: 16-19 kDa; IP: 5-6) were also lower than those in mammals (molecular mass: 40 kDa; IP: 9.4).

The mRNA of *LV-AT_1_R* was detected in all of the examined tissues. Eight weeks of long-term low salinity stress did not affect the expression level of *LV-AT_1_R* in the eyestalk, hepatopancreas, or muscle. During short-term low salinity stress, *LV-AT_1_R* was downregulated in the eyestalk at 12 h and 24 h. In the hepatopancreas and muscle, the downregulation was observed at 3 h and 24 h, respectively. The regulatory mechanism of AT_1_R in invertebrates is unknown, and the information is limited to some vertebrate species. AT_1_R receptors belong to the family of G protein-coupled receptors (GPCRs). Angiotensin II and angiotensin III stimulate *AT_1_R* receptors, and these receptors are inhibited by members of the sartan family such as losartan (38). Tyrosine^4^ and phenylalanine^8^ side chains are the key amino acids in angiotensin II that activate *AT_1_R* receptors (39). Angiotensin II and angiotensin III are the main effector peptides in the RAS pathway. The role of angiotensin II and angiotensin III in stimulating drinking, decreasing urine output, and perfusate flow rate in the kidney is well documented in fishes and mammals (38). In semiterrestrial crabs (*Chasmagnathus granulatus*), water deprivation elevated the level of angiotensin II neuropeptide in the brain and optic lobes (40). Two hours after water deprivation, the immunoreactivity of angiotensin II neuropeptide increased in the central body and decreased in the olfactory neuropil, indicating that angiotensin II regulates several neuronal activities during water shortages (40). In the clam worm *Perinereis* sp., angiotensin III and angiotensin II regulate body weight under different osmotic conditions. Angiotensin II and angiotensin III suppress net body fluid loss under hyperosmotic stress. Under hypoosmotic stress, they enhance net body fluid gain. Under drying conditions, angiotensin II suppressed body weight loss, but angiotensin III did not. These results showed that these two peptides regulate body fluid volume *via* the angiotensin II receptor in various ways (41).

### Identification, characterization, and expression analysis of *LV-RR*

Sequence homology and phylogenetic analysis showed that the LV-RR protein had the highest degree of homology with renin receptors in *P. monodon* (94.74%), *P. chinensis* (93.50%), and *P. japonicus* (88.85%). The structure and sequences of the LV-RR protein in 18 various species were compared. The LV-RR protein in crustaceans consists of 323 amino acid residues. In crustaceans, the molecular mass and estimated IP were approximately 4.95 and 35 kDa, respectively. The LV-RR protein in insects and fishes was relatively larger than that in crustaceans and mammals. Unlike other elements of the RAS pathway, the *RR* gene is extremely conserved among species, and RR orthologs are found in several species from mammals to insects and crustaceans (42). The RR protein in mammals consists of approximately 350 amino acids and has a single transmembrane domain and a short cytoplasmic domain that has no intrinsic kinase activity. In mammals, the percent identity among human, rat, and mouse RR proteins is approximately 95% for the nucleotide sequence and over 80% for the amino acid sequences, showing that RR protein is a highly conserved protein (43).

The *LV-RR* mRNA was expressed in all of the examined tissues. The expression level of *LV-RR* in the eyestalk, hepatopancreas, and muscle was not affected by long-term low salinity stress. Short-term low salinity stress significantly downregulated the expression level of *LV-RR* in the eyestalk at 12 h and 24 h. In the hepatopancreas, the downregulation was recorded at 96 h. In the muscle, the expression of *LV-RR* significantly decreased at 3 h, 24 h, and 72 h of short-term low salinity stress. The RR protein is a multifunctional protein existing in different molecular forms, and its regulatory mechanism is very complicated. In addition to the RAS pathway, the activation of RR is triggered by other signaling pathways, such as the MAP kinase p38-heat shock protein 27 cascade and the PI3K-p85 pathway (44). Renin is an important modulator of the RAS pathway. Renin is the first rate-limiting step of the RAS, and its suppression directly affects the whole RAS pathway (45). Moreover, renin itself is modulated by several components of the RAS pathway and other pathways, including intracellular cAMP (stimulatory), Ca^2+^ (inhibitory), and cyclic guanosine monophosphate signaling pathways (30).

## Conclusion

In the present study, four key genes (*LV-ACE*, *LV-APN*, *LV-AT_1_R*, and *LV-RR*) in the RAS pathway were cloned and characterized, and their expression patterns were investigated in different tissues (eyestalk, hepatopancreas, and muscle) under different salinity conditions. The *LV-ACE*, *LV-APN*, *LV-AT_1_R*, and *LV-RR* genes encode 666, 936, 175, and 323 amino acids, respectively. All four genes were expressed in all the examined tissues. In terms of the RAS pathway and its related genes, the eyestalk was the most sensitive tissue. Low salinity stress downregulated the expression levels of *LV-ACE*, *LV-APN*, *LV-AT_1_R*, and *LV-RR* in the eyestalk. Other tissues also displayed a similar trend, and the downregulation of these four genes indicates that the RAS pathway was suppressed under low salinity. The results of this study provide valuable information regarding the role of the RAS pathway in nonvertebrate species and the mechanism of salinity adaptation in *L. vannamei*. Further studies are required to reveal the regulatory mechanism (i.e., eyestalk ablation) and functional analysis (i.e., RNA interference and *in situ* hybridization) of the RAS pathway and its related genes in *L. vannamei*.

## Data availability statement

The datasets presented in this study can be found in online repositories. The names of the repository/repositories and accession number(s) can be found in the article/**Supplementary Material**.

## Author contributions

AF: manuscript writing, data analysis. YL: conducting experiments, data analysis. CX: conducting experiments, validating the data, and proofreading the manuscript. XW: experimental design, validation of the data, proofreading of the manuscript. EL: funding acquisition, supervision, experimental design, validation of the data, proof reading of the manuscript. All authors contributed to the article and approved the submitted version.
