# Supplementary material for: The role of the renin-angiotensin system (RAS) in salinity adaptation in Pacific white shrimp (Litopenaeus vannamei)
Source: Front Endocrinol (Lausanne). 2022 Dec 15;13:1089419. doi: 10.3389/fendo.2022.1089419 (PMC9798321; doi:10.3389/fendo.2022.1089419)
Supplement: Supplementary file 1 [file DataSheet_1.docx]

The 3D protein structure of LV-ACE is shown in Supplementary Figure 1A. Using the SOPMA server, we showed that the LV-ACE protein contains 54.37% α helix, 4.29% β turn, 33.28% random coil, and 8.13% extended strand (Supplementary Figure 1B). The SignalP 5.0 tool predicted the signal peptide sites (Supplementary Figure 1C). The cleavage site was located between amino acids 14 and 15. The LV-ACE protein contains a peptidase_M2 domain located from amino acids 32-680 (Supplementary Figure 1D).


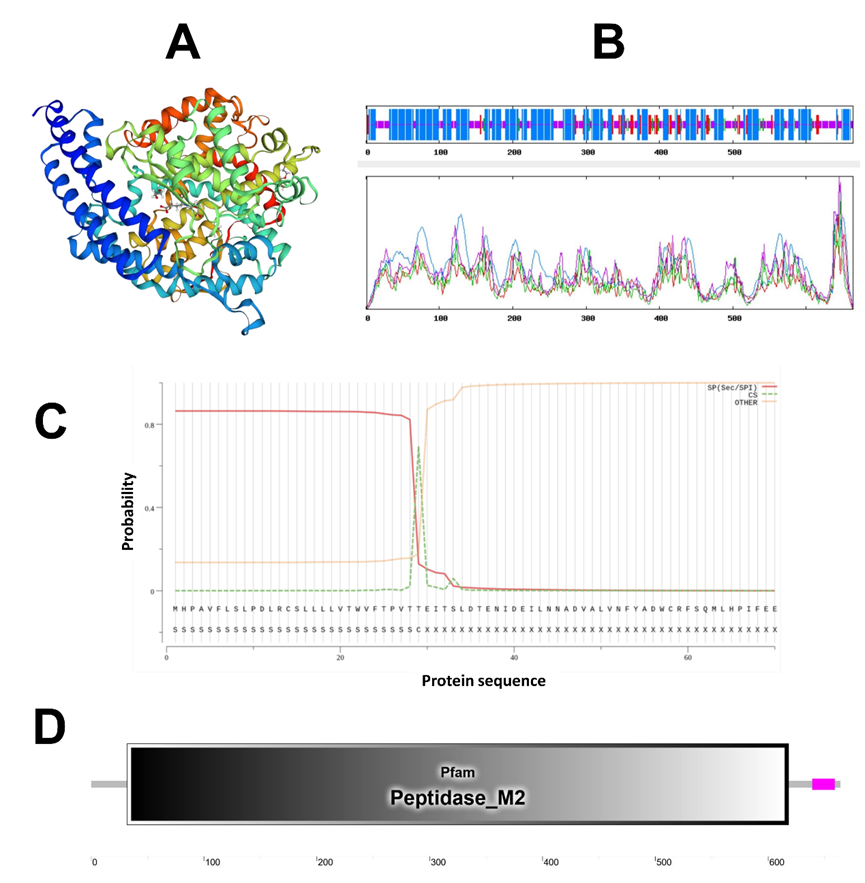


**Supplementary Figure 1.** (A): The three-dimensional (3D) protein structure of LV-ACE. (B): Analysis of the secondary structure. The a-helix (blue), extended strand (red), random coil (purple), and b-turn (green). (C): Signal peptide prediction in the LV-ACE coding region. (D): Schematic description of the domain topologies of the LV-ACE coding region.

The 3D structure of the LV-APN protein is presented in Supplementary Figure 2A. The LV-APN protein consisted of 45.19% α helix, 4.06% β turn, 36.22% random coil, and 14.53% extended strand (Supplementary Figure 2B). The cleavage site for the LV-APN protein was predicted to be amino acids 15-16 (Supplementary Figure 2C). SMART analysis revealed that the LV-APN protein contained three domains (Supplementary Figure 2D): the d1hs6a2 domain (from amino acids 40-247), the Peptidase_M1 domain (from amino acids 259-503), and the ERAP1_C domain (from amino acids 587-908).


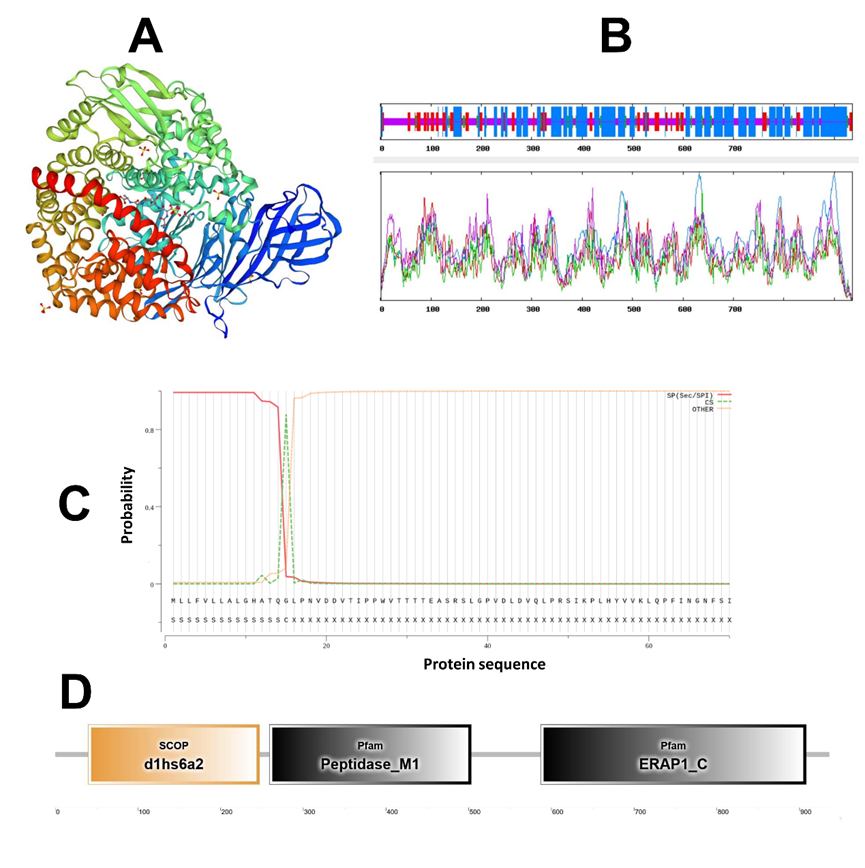


**Supplementary Figure 2.** (A): The three-dimensional (3D) protein structure of LV-APN. (B): Analysis of the secondary structure. The a-helix (blue), extended strand (red), random coil (purple), and b-turn (green). (C): Signal peptide prediction in the LV-APN coding region. (D): Schematic description of the domain topologies of the LV-APN coding region.

Supplementary Figure 3A shows the 3D structure of the LV-AT_1_R protein. The secondary structure of the LV-AT_1_R protein includes 32% α helix, 1.14% β turn, 49.14% random coil, and 17.71% extended strand (Supplementary Figure 3B). No signal peptide site was predicted for the LV-AT_1_R protein (Supplementary Figure 3C). The LV-AT_1_R protein contains an angiotensin II, type I receptor-associated protein domain (AGTRAP) on amino acids 1-167 (Supplementary Figure 3D).


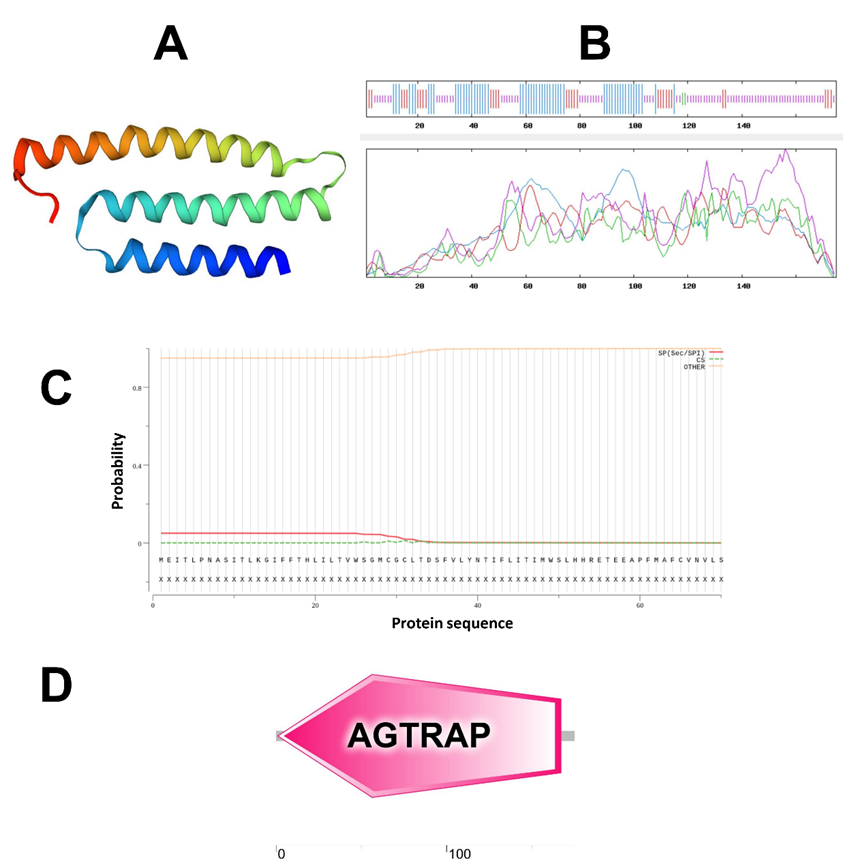


**Supplementary Figure 3.** (A): The three-dimensional (3D) protein structure of LV-AT_1_R. (B): Analysis of the secondary structure. The a-helix (blue), extended strand (red), random coil (purple), and b-turn (green). (C): Signal peptide prediction in the LV-AT_1_R coding region. (D): Schematic description of the domain topologies of the LV-AT_1_R coding region.

The 3D structure of the LV-RR protein is presented in Supplementary Figure 4A. The SOPMA server revealed that the RR protein contained 43.03% α helix, 5.26% β turn, 35.60% random coil, and 16.10% extended strand (Supplementary Figure 4B). The cleavage site was located between amino acids 20 and 21 (Supplementary Figure 4C). The LV-RR protein contains a renin receptor-like protein domain (Renin_r) on amino acids 229-322 (Supplementary Figure 4D).


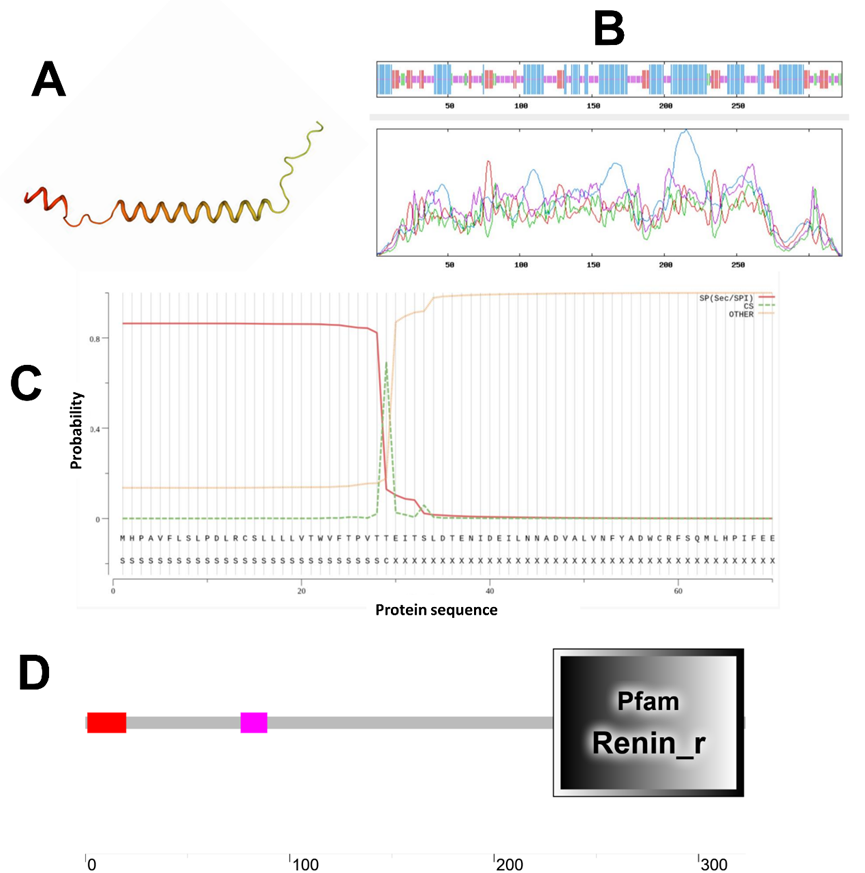


**Supplementary Figure 4.** (A): The three-dimensional (3D) protein structure of LV-RR. (B): Analysis of the secondary structure. The a-helix (blue), extended strand (red), random coil (purple), and b-turn (green). (C): Signal peptide prediction in the LV-RR coding region. (D): Schematic description of the domain topologies of the LV-RR coding region.


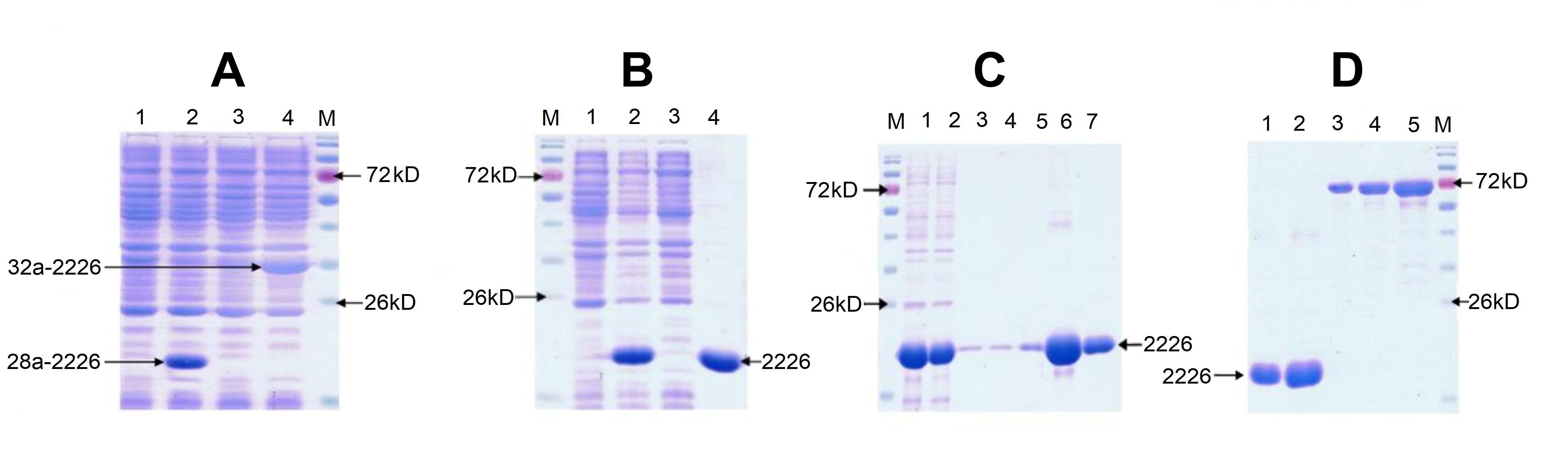


**Supplementary Figure 5.** Purification and expression of LV-ACE protein. (A): The ligated protein fragments with prokaryotic expression plasmids pET28a and pET32a. (B): Solubility determination of LV-ACE recombinant protein by ultrasonic disruption of bacteria. (C): SDS‒PAGE for purification of LV-ACE recombinant protein. (D): SDS‒PAGE for quantification of LV-ACE recombinant protein.
