# Supplementary material for: The role of the renin-angiotensin system (RAS) in salinity adaptation in Pacific white shrimp (Litopenaeus vannamei)
Source: Front Endocrinol (Lausanne). 2022 Dec 15;13:1089419. doi: 10.3389/fendo.2022.1089419 (PMC9798321; doi:10.3389/fendo.2022.1089419)
Supplement: Supplementary file 2 [file DataSheet_2.docx]

**The sequences of the amplified products for LV-ACE gene**

> LV-ACE (Present study)

CCATGTTCGTGTTGTGGATGTGTTTGTTGTACGGTGTGGCTGCGGTGCCACAGGAGTCTCCAGCCCAACTAGCAGGGCAAGCACACGCCGAGTCCGTGAGTCTGGAGGAGGAAGCCAGGTCTTTCCTGGACGAGCTCGACGCCCGCGACTCCGAGGAGTGCTCCGCCGTCAGCTTCGCCTCCTGGAACTACGATTCCGACATCACCGAAAAGCACAAGGCTGAGAAGGCCGCCGCCCAGCTCACGCACGCCGCCTGGGAGAAGGAGGCGTGGCAGCGCGTGCAGAAGTGGAACGGCCGCTGGGAGGCGCTCACCGACCCCGCCCTCAGAAGGCAGTTCAAGTTCCGGTCCATCCTGGGCACGGCGGCGCTGCCCAAGCAGGAGCTCGAGAAGTACAACAGCCTGGAGACGGACATGTCCACCATCTACAGCACGGCCAAGATCTGCGACTACCACAGGCACAAGGTGTGCAACCTGACCCTCGAACCAGACCTGACGAGGATCCTGCGCACGAGCCGCGACTACGGCGAGCTGGCGCACGTGTGGAAGCAGTGGCGCTACAACTCCGGCCGGAGGATCAGGGACCACTACCGCCTCTTCGTCAACATCGCCAACAAGGCCGCCAAGCTCAACGGTTTCGACAACATGGGGGACATGTGGCTTTACCCGTACGAGTCCGAGACCTTCCGGCAGGACGTTGCTGAGCTGTGGGAGCAACTGAAGCCCTTGTACCAGCAGCTGCACGCCTACGTTAGGAGGAAGTTGCGAGCGAAATATGGAGAGCAGCACATCAGCAAGCGAGGGCCCATTCCCGCGCATCTGCTGGGGAACATGTGGTCGCAGTCTTGGGGCGAAATATATGACCTTCTGATCCCTTACCCTGGGAAGACCTCAGTTGATGTCACGCCGAACATGAAAAGCCAGGGCTACACTCCACGGAGGATGTTCGAGCTCTCGGAGGAGTTCTTCGTGTCGCTGAACCTGACCCGGATGCCGCCCGAGTTCTGGGAGCACTCCATCATCGAGAAGCCGAACGGGCGGGAGCTGATCTGCCACGCCTCCGCTTGGGACTTCTGCAACGGCAAAGACTTCAGAATAAAGCAGTGCACAGATGTCACCATGACTGACTTGATCACAGTGCACCACGAAATGGGCCACATCCAATACGACCTGCAGTACAAGCACCTGCCGCTCGTGTTCCGTGGCGGGGCTAATCCAGGTTTCCATGAGGCCGTTGGAGATGTGTTGGCTCTGAGTGTGGCCACGCCAAAACATCTTCAGAAGATTGGCTTGCTGGAAACTGTTGAAGAGGACAATGAAGCCGACATCAATTACCTCATGAGCATAGCCTTGGACAAGATCACTTTCTTACCTTTTGGTTATCTGATGGACCTGTGGCGCTGGGGAGTGTTCAGCGGGGAAACCAAAGAGGAGGATTGGAACTGCGCCTGGTGGGCCTTGAGATATAATCTGCAGGGCATAAAGCCTCCTGTGAGACGATCAGAGTATGATTTTGATCCTGGGGCTAAGTATCACATCCCAGCCAACGTTCCCTACATCAGGTACTTCGTCAGCTTCGTGGTTCAGTTTCAGTTCCACAAAGCACTCTGCATGAAGGCTGGGGAATATGACCCTCTAGACCCCTCAAAACCGCTCCACAAGTGTGACATCTATCAGTCCACGGAAGCAGGAAATGCACTCGGGGACATGTTGCAGCTAGGCTCATCGAAGCCCTGGCCAGATGCCCTCGAGGCGCTGACGGGCACCAGGAGGATGGACGCCTCCATCATCAGGGAATATTTCAAGCCCCTGGAGGTCTGGCTCACGGAGAACAACGAGAGACACCAGGAGTTTGTGGGCTGGGAAGGGGAGAGCGACTTCTGTTCCTCGGACTCCCCCGCGCGCCACGAGCCCGTCGGCGAGCCCTCCTCGGCGGCCGCGGTCTCCCCGGGCCCGCTCGCGCTGGCTCTCGTGGCGCTTCTGGTCCATCGAACCCAACAATAATTTCCAGTTTAGAATAGGAATGTACATTTTTTAGATCTACGGAAATATTGGAGATTTCTATGATAAGTGGGTGAGATGATGTGCAGTGATTTGTTGAATAGTCTAGTAAGTGTTTTGTTGGAGAAACAACAAATTCATGGTTTAACCGTTTCTTTTTCAATTTCAATAAATGTATTATTTCGGAAGGATGAAAGGATCTATTATATTTTTTGCCGTGGAATGTGCATTACACGTGTGTATGAGAGACTTACTGTATGTATAAATACTGAATTAAAAAATGCGACTTAAAGGGATTGTGTGTACTCTATATATTTTAAATAAAG

**The protein sequences that used for bio informatics**

**>> Protein sequences of Present study (angiotensin-converting enzyme-like [Penaeus vannamei])**

MFVLWMCLLYGVAAVPQESPAQLAGQAHAESVSLEEEARSFLDELDARDSEECSAVSFASWNYDSDITEKHKAEKAAAQLTHAAWEKEAWQRVQKWNGRWEALTDPALRRQFKFRSILGTAALPKQELEKYNSLETDMSTIYSTAKICDYHRHKVCNLTLEPDLTRILRTSRDYGELAHVWKQWRYNSGRRIRDHYRLFVNIANKAAKLNGFDNMGDMWLYPYESETFRQDVAELWEQLKPLYQQLHAYVRRKLRAKYGEQHISKRGPIPAHLLGNMWSQSWGEIYDLLIPYPGKTSVDVTPNMKSQGYTPRRMFELSEEFFVSLNLTRMPPEFWEHSIIEKPNGRELICHASAWDFCNGKDFRIKQCTDVTMTDLITVHHEMGHIQYDLQYKHLPLVFRGGANPGFHEAVGDVLALSVATPKHLQKIGLLETVEEDNEADINYLMSIALDKITFLPFGYLMDLWRWGVFSGETKEEDWNCAWWALRYNLQGIKPPVRRSEYDFDPGAKYHIPANVPYIRYFVSFVVQFQFHKALCMKAGEYDPLDPSKPLHKCDIYQSTEAGNALGDMLQLGSSKPWPDALEALTGTRRMDASIIREYFKPLEVWLTENNERHQEFVGWEGESDFCSSDSPARHEPVGEPSSAAAVSPGPLALALVALLVHRTQQ

>XP_047478273.1 angiotensin-converting enzyme-like [Penaeus chinensis] MSTIYSTAKICDYHRTKVCNLTLEPDLTRILRTSRDYGELAHVWKQWRYKSGRRIRDHYRLFVNIANKAA KLNGFDNMGDMWLYPYESDTFRQDVGELWEQLKPFYQQLHAYVRRKLREKYGEQHISQRGPIPAHLLGNM WSQSWGEIYDLLIPYPGKTSVDVTPNMKTKGYTPRRMFELSEEFFVSLNLTRMPPEFWEHSIIEKPKGRE LICHASAWDFCNGKDFRIKQCTDVTMTDLITVHHEMGHIQYYLQYKHQPNVFRAGANPGFHEAVGDVLAL SVATPKHLQKIGLLDNVEEDNEADINYLMSIALDKITFLPFGYLMDLWRWGVFSGKTKEENWNCAWWDLR YNLQGIKPPVRRSEYDFDPGAKYHIPANVPYVRYFVSFVIQFQFHKALCMKAGQYDPLDPSKPLHKCDIY QSTEAGNALGDMLQMGSSKPWPDALEALTGTRKMDASIIREYFKPLEVWLTENNERHQEFVGWEGENDFC SSDSPARHEPVGEPSSGAAVSPGLLVVVLMAFLLQQTHQ

>XP_042873558.1 angiotensin-converting enzyme-like isoform X1 [Penaeus japonicus] MLVLWMCLLYGVAAVPQDSPAQLAGQANAESLSLEEEARNFLNELDGRDSEECSAVSFASWNYDSDITEK HKAEKAAAQLTHAAWEKEAWQRVRKWNGRWEALTDPFLRRQFKFRSILGTAALDKPELEKYNSLETDMST IYSTAKICDYHKHKMCNLTLEPDLTRTLRTSRDYDELAHVWKQWRYNSGRRIRDHYRLFVNIANKAAKLN GFDNMGDMWLYPYESETFRQDVAGLWEQLKPLYQQLHAYVRRKLREKYGSQHISKRGPIPAHLLGNMWSQ SWGEIYDLLIPYPGKTSVDVTPNMKNQGYTPRKMFELSEEFFVSLNLTRMPPEFWEHSIIEKPKGRDLIC HASAWDFCNGKDFRIKQCTDVTMTDLITVHHEMGHIEYYLQYEHQPLVFRSGANPGFHEAVGDVLALSVA TPKHLKKIGLLESVEEDDEADVNYLMSIALDKIAFLPFGYLMDLWRWGVFSGETKEENWNCAWWDLRYKL QGIKPPVKRSEYDFDPGAKYHIPANVPYIRYFVSFVVQFQFHKALCMKAGEYDPLDPSKPLHKCDIYQST EAGNALGDMLQLGSSKPWPDALEALTGTRRMDASIIREYFKPLEVWLTENNERHQEFVGWEGENDFCSSD SPARHEPVGEPSAATALSPGMLVLALMALLFRQSLQ

>CAX63316.1 TPA: putative angiotensin converting enzyme precursor, partial [Carcinus maenas] MCVWVCCLALVAAIPQDSASTGEGGTSMDEMESKARSFLQEMDMRGSKECTQATMASWNYASDITEKHRK EKAAAQIKYATWEKEVWEKVKEWDGQWQQLGDPFLKRQFKFMSILGTAALPKKELEKYNNLVTEMSTIYS TAKICGYKKNSSCKLSLEPDLTKILRTSRDYGELEHVWKMWRTASGRRMRNLYQQFVKLANKAAKLNGFE NMGEMWIYPYESDTFRTDLHHLYMQLKALYQQLHAYVRRKLRERYGEEHVSQRGPIPAHLMGNMWSQSWA EIYDLAIPYPGKTSVDVTPQMVAQGYDARRMFELSEEFFVSLNLTRMPQGFWQNSIIEKPQGRELVCHAS AWDFCDGKDVRIKQCTEVTMKDLITVHHEMGHIEYYLQYSHQPKLFREGANPGFHEAVGDVLALSVATPK HLHKVGLLDQVEDNHEVDINFLMNMALDKITFLPFGYLMDLWRWDVFSGKAATKDWNCFWWKLRYEFQGI KPPVMRSEYDFDPGAKYHIGANVPYIRYFVSFVVQFQFHKALCMKAGQYDPLDPSKPLHKCDIYESTEAG NILGDMLQMGSSKEWPDAMEVLTGGRVMDASPIREYFKPLEMWLKEDNEKHGEFIGWESDETYCSSEGMS EEATSEPTSAASCCSPASLLLLLVVALFSLNH

>XP_010082463.1 PREDICTED: LOW QUALITY PROTEIN: angiotensin-converting enzyme [Pterocles gutturalis] MSPLTVQVEASLEEQNFTELWGKKAKELYSNIWSNFSDPQLRKIIGSIQTLGPSNLPLEKREQYNTILSN MDKIYSTAKVCLANGTCWDLEPDLSDIMATSRSYKKLLYAWEGWHNAAGNPLRAPYEEFVKLSNEAYQMD GFEDTGSYWRSWYDSPSFEDDLEHLYKQLEPLYLNLHAFVRRKLYNRYGPKYINLRGPIPAHLLGNMWAQ QWNNIYDLMVPYPEKPNLDVTSTMVQQGWNATHMFRVSDEFFTSLGLLEMPPEFWEKSMLEKPTDGREVV CHASAWDFYNRKDFRIKQCTTVTMEQLFTVHHEMGHVQYYLQYKDQPVSFRSGANPGFHEAIGDVMSLSV STPSHLKKIGLLSSATEDAESNINYLLKMALEKIAFLPFGYLIDQWRWNVFNGRTPPNRYNYDWWYLRTK YQGICAPVSRDETNFDPGAKYHIPGNTPYIRYFASFILQFQFHKALCQAANHNGPLHTCDIYMSKEAGAK LREVLKAGSSKSWQEILFNLTGTDKMDAGALLEYFSPVTQWLKEQNNKTNEVLGWPEFDWRPPVPEGYPE GIDKIADEAQAKEFLSEYNSTAEAVWNAYTEASWTYNTNITDHNKEIMLEKNLAMSKHTLEYGMRARQFD TSDFQDQSVTRILKKLSVIEKAALPENELKEYNTLLSDMETTYSVAKVCRESKTCHPLDPDLTDILANSR DYDELLFAWKGWRDASGKKIKNSYKRYVELSNKAAVLNGYMDNGAFWRSLYETPTFEEDLERLYLQLQPL YLNLHAYVRRALHKKYGAERINLKGPIPAHLLGNMWAQSWSSIFDLVTPFPDATKVDATPAMKQQGWTAK RMFEESDHFFTSLGLIPMPQEFWDKSMIEKPADGREVVCHASAWDFYNRKDFRIKQCTVVNMDDLITVHH EMGHVQYFLQYMDQPISFRDGANPGFHEAIGDVMALSVSTPKHLHSIKLLDQVTDNQESDINYLMSIALD KIAFLPFGYLMDQWRWKVFDGRIKEDEYNQQWWNLRLKYQGLCPPTPRSEDDFDPGAKFHIPANVPYIRY FVSFVIQFQFHQALCAAAGHTGPLHTCDIYQSKEAGKLLGDALKLGFSKPWPEAMELITGQPNMSADALM SYFEPLMTWLVKENERNGEVLGWPEYSWTPYAATAAQPGSSRTDFLGMSLTSKEATAGVLLALALVFLI TTIFLGIKFFSERRSQTFKSSSEMELK

>XP_010012983.1 PREDICTED: angiotensin-converting enzyme [Nestor notabilis] MEVWGKKAKELYGSIWSNFSDTQLRKIISSIQTLGPSNLPLDKREQYNTILSDMDKIYSTAKVCLANSTC WELEPDISDIMATSRSYKKLLYAWEGWHNAAGNPLRAKYEEFVQLSNEAYQMDGFEDTGSYWRSWYDSAS FEDDLEHLYNQVEPLYLNLHAFVRRKLYDRYGSKYINLKGPIPAHLLGNMWAQQWNNIYDLMIPYPDKPN LDVTSTMVQQGWNATHMFRVSEEFFTSLGLLEMPPEFWDKSMLEKPADGREVVCHASAWDFYNRKDFRIK QCTTVTMEQLFTVHHEMGHIQYYLQYKDQPVSFRSGANPGFHEAIGDVMSLSVSTPSHLKEIGLLNSAAE DTESNINYLLKMALEKIAFLPFGYLIDQWRWNVFSGRTPPSRYNYDWWYLRTKYQGICAPISRNESNFDP GAKYHIPGNTPYIREVLKAGSSKSWQEILFNLTGTDKMDAGALLEYFSPVTEWLQEQNNKTNEVLGWPEF DWRPPIPEGYPEGIDKISDEAQAKEFLAEYNSTAEAVWNAYTEASWAYNTNITDHNKEIMLEKNLAMSRH TLEYGMRARQFDPSDFQDQSVTRILRKLSVIERAALPEDELKEYNTLLSDMETTYSIAKVCRENKTCHPL DPDLTDIMATSRDYDELLFVWKGWRDASGKQIKNNYKRYVELSNKAAVLNGYSDNGAFWRSLYETSTFEE DLERLYLQLQPLYLNLHAYVRRALYRKYGGEHINLKGPIPAHLLGNMWAQSWSNIFDLVMPFPDATKVDA TPAMKQQGWTPKRMFEESDRFFTSLGLIPMPEEFWDKSMIEKPADGREVVCHASAWDFYNRKDFRIKQCT VVNMDDLITVHHEMGHVQYFLQYMDQPISFRDGANPGFHEAVGDVMALSVSTPKHLHSINLLDQVTDNTE SDINYLMSIALDKIAFLPFGYLMDQWRWKVFDGRIKEDEYNQEWWNLRLKYQGLCPPTPRSEDDFDPGAK FHIPANVPYIRYFVSFVIQFQFHQALCAAAKHTGPLHTCDIYQSKEAGNILGEALKLGFSKPWPEAMELI TGQPNMSADALMSYFEPLMTWLVNENEKNEEVLGWPEYSWTPYTATTAQAGSSRTDFLGMSLASNQATAG SWVLLALALVFLITTIYLGVKFSSARRKTFKSSSEMELK

>XP_008944127.1 PREDICTED: angiotensin-converting enzyme-like, partial [Merops nubicus] PGNMWAQSWSNIFDLVIPFPNATKVDATPAMKKQGWTPRKMFEVSDHFFTSLGLIPMPEEFWNKSMIEKP ADGREVVCHASAWDFYNRKDFRIKQCTVVNMDDLITVHHEMGHVQYFLQYMDQPISFRDGANPGFHEAIG DVMALSVSTPKHLHSINLLDQVTDNEESDINYLMSIALDKIAFLPFGYLMDQWRWKVFDRRIKEDEYNQQ WWNLRMKYQGLCPPAPRSEDDFDPGAKFHIPANVPYIRYFVSFVIQFQFHQALCAVAKHQGPLHKCDIYK SKDAGSILGKALKLGFSRPWPEAMMLITKQPNMSADALMSYFEPLMTWLVKENEKNGEVLGWPEYSWTPY TATPAQAVSDHADFLGMSLTSHQATAGGWVLLALALVFLVTTLFFGVKFFSSRRKAFKSSSEMELK

>XP_009491429.1 PREDICTED: angiotensin-converting enzyme [Pelecanus crispus] MDKIYSTAKVCLANGTCWDLEPDISDIMATSRSYKKLLYAWEGWHNAAGNPLRTKYEEFVKLSNEAYQMD GFKDTGSYWRSWYDSDTFEDDLEQLYKQLEPLYLNLHAFVRRKLYDRYGPKYVNLKGPIPAHLLGNMWAQ QWNNIYDLMVPYPAKPNLDVTSTMVQQEWNATRMFRVSEEFFTSLGLLEMPPEFWNKSMIEKPTDKREVV CHASAWDFYNRKDFRIKQCTTVTMEQLFTVHHEMGHVQYYLQYKDQPVPFRAGANPGFHEAIGDVMALSV STPSHLKKIGLLSSATEDAESNINYLLKMALEKIAFLPFGYLIDQWRWNVFNGRPPRSRYNYDWWYLRTK YQGICAPVPRNESNFDPGAKYHIPANTPYIRYFVSFIIQFQFHKALCQAANHSGPLHTCDIYMSKQAGDK LRELLKAGSSKSWQEIMFNLTATNKMDAGPLLEYFSPVTKWLQEQNKKTNEVLGWPEFDWHPPIPEGYPE GIEKIADEAQAKAFLSEYNSTAEAVWNAYTEASWAYNTNITDHNKEIMLQKNLAMSKHTLEYGRRARQFD TSNFQDQSVTRILKKLSVIERAALPENELKEYNTLLSDMETAYSVAKVCKENNVCHPLDPDLTDIMATSR DYDELLFAWKGWRDASGKKIKNNYKRYVELSNKAAVLNGYTDNGAYWRSLYETPTFEEDLERLYLQLQPL YLNLHAYVRRALYKKYGAEHINLKGPIPAHLLGNMWAQSWSNIFDLVTPFPDATKVDATPAMKKQGWTPK KMFEESDHFFTSLGLIPMPQEFWNKSMIEKPADGREVVCHASAWDFYNRKDFRIKQCTVVNMDDLITVHH EMGHVQYFLQYKDQPISFRDGANPGFHEAIGDVMALSVSTPKHLHSIKLLDQVTDNTESDINYLMSIALD KIAFLPFGYLMDQWRWKVFDGRIKEDEYNQQWWNLRMKYQGLCPPTPRSEDDFDPGAKFHIPANVPYIRY FVSFVIQFQFHQALCAAARHTGPLHKCDIYQSKEAGKILGEALKLGFSKPWPEAMRLITGQPNMSADALM SYFEPLMTWLTTENKKNGEVLGWPEYSWTPYAAIPDQASSSRMDFLGMSLASNQATAGSWVLLTLALVFL ITTISLGVTFFLARKKVSKSSSEMELK

>KAI4050971.1 angiotensin I converting enzyme [Homo sapiens] MGQGWATAGLPSLLFLLLCYGHPLLVPSQEASQQVTVTHGTSSQATTSSQTTTHQATAHQTSAQSPNLVT DEAEASKFVEEYDRTSQVVWNEYAEANWNYNTNITTETSKILLQKNMQIANHTLKYGTQARKFDVNQLQN TTIKRIIKKVQDLERAALPAQELEEYNKILLDMETTYSVATVCHPNGSCLQLEPALKFSELPLQAEVS

>NP_036676.1 angiotensin-converting enzyme precursor [Rattus norvegicus] MGAASGQRGRWPLSPPLLMLSLLLLLLLPPSPAPALDPGLQPGNFSADEAGAQLFADSYNSSAEVVMFQS TAASWAHDTNITEENARLQEEAALINQEFAEVWGKKAKELYESIWQNFTDQKLRRIIGSVQTLGPANLPL TQRLQYNSLLSNMSRIYSTGKVCFPNKTATCWSLDPELTNILASSRNYAKVLFAWEGWHDAVGIPLKPLY QDFTALSNEAYRQDGFSDTGAYWRSWYESPSFEESLEHLYHQVEPLYLNLHAFVRRALHRRYGDKYINLR GPIPAHLLGDMWAQSWENIYDMVVPFPDKPNLDVTSTMVQKGWNATHMFRVAEEFFTSLGLSPMPPEFWA ESMLEKPADGREVVCHASAWDFYNRKDFRIKQCTRVTMDQLSTVHHEMGHVQYYLQYKDLHVSLRRGANP GFHEAIGDVLALSVSTPAHLHKIGLLDRVANDIESDINYLLKMALEKIAFLPFGYLVDQWRWGVFSGRTP PSRYNYDWWYLRTKYQGICPPVARNETHFDAGAKFHIPSVTPYIRYFVSFVLQFQFHQALCKEAGHQGPL HQCDIYQSTKAGAKLQQVLQAGCSRPWQEVLKDLVGSDALDASALMEYFQPVSQWLQEQNQRNGEVLGWP EYQWRPPLPDNYPEGIDLETDEAKANRFVEEYDRTAKVLWNEYAEANWHYNTNITIEGSKILLQKNKEVS NHTLKYGTWAKTFDVSNFQNSTIKRIIKKVQNVDRAVLPPNELEEYNQILLDMETTYSVANVCYTNGTCL SLEPDLTNIMATSRKYEELLWVWKSWRDKVGRAILPFFPKYVDFSNKIAKLNGYSDAGDSWRSSYESDDL EQDLEKLYQELQPLYLNLHAYVRRSLHRHYGSEYINLDGPIPAHLLGNMWAQTWSNIYDLVAPFPSAPSI DATEAMIKQGWTPRRIFKEADNFFTSLGLLPVPPEFWNKSMLEKPTDGREVVCHASAWDFYNGKDFRIKQ CTSVNMEELVIAHHEMGHIQYFMQYKDLPVTFREGANPGFHEAIGDVLALSVSTPKHLHSLNLLSSEGSG YEHDINFLMKMALDKIAFIPFSYLIDQWRWRVFDGSITKENYNQEWWSLRLKYQGLCPPVPRSQGDFDPG SKFHVPANVPYIRYFISFIIQFQFHEALCRAAGHTGPLYKCDIYQSKEAGKLLADAMKLGYSKQWPEAMK IITGQPNMSASAIMNYFKPLTEWLVTENRRHGETLGWPEYTWTPNTARAEGSLPESSRVNFLGMYLEPQQ ARVGQWVLLFLGVALLVATVGLAHRLYNIHNHHSLRRPHRGPQFGSEVELRHS

>NP_001268748.1 angiotensin-converting enzyme isoform 3 precursor [Mus musculus] MGAASGQRGRWPLSPPLLMLSLLVLLLQPSPAPALDPGLQPGNFSPDEAGAQLFAESYNSSAEVVMFQST VASWAHDTNITEENARRQEEAALVSQEFAEVWGKKAKELYESIWQNFTDSKLRRIIGSIRTLGPANLPLA QRQQYNSLLSNMSRIYSTGKVCFPNKTATCWSLDPELTNILASSRSYAKLLFAWEGWHDAVGIPLKPLYQ DFTAISNEAYRQDDFSDTGAFWRSWYESPSFEESLEHIYHQLEPLYLNLHAYVRRALHRRYGDKYVNLRG PIPAHLLGDMWAQSWENIYDMVVPFPDKPNLDVTSTMVQKGWNATHMFRVSEEFFTSLGLSPMPPEFWAE SMLEKPTDGREVVCHASAWDFYNRKDFRIKQCTRVTMEQLATVHHEMGHVQYYLQYKDLHVSLRRGANPG FHEAIGDVLALSVSTPAHLHKIGLLDHVTNDIESDINYLLKMALEKIAFLPFGYLVDQWRWGVFSGRTPP SRYNFDWWYLRTKYQGICPPVARNETHFDAGAKFHIPNVTPYIRYFVSFVLQFQFHQALCKEAGHQGPLH QCDIYQSAQAGAKLKQVLQAGCSRPWQEVLKDLVGSDALDAKALLEYFQPVSQWLEEQNQRNGEVLGWPE NQWRPPLPDNYPEGIDLETDEAKADRFVEEYDRTAQVLLNEYAEANWQYNTNITIEGSKILLEKSTEVSN HTLKYGTRAKTFDVSNFQNSSIKRIIKKLQNLDRAVLPPKELEEYNQILLDMETTYSLSNICYTNGTCMP LEPDLTNMMATSRKYEELLWAWKSWRDKVGRAILPFFPKYVEFSNKIAKLNGYTDAGDSWRSLYESDNLE QDLEKLYQELQPLYLNLHAYVRRSLHRHYGSEYINLDGPIPAHLLGNMWAQTWSNIYDLVAPFPSAPNID ATEAMIKQGWTPRRIFKEADNFFTSLGLLPVPPEFWNKSMLEKPTDGREVVCHPSAWDFYNGKDFRIKQC TSVNMEDLVIAHHEMGHIQYFMQYKDLPVTFREGANPGFHEAIGDIMALSVSTPKHLYSLNLLSTEGSGY EYDINFLMKMALDKIAFIPFSYLIDQWRWRVFDGSITKENYNQEWWSLRLKYQGLCPPVPRSQGDFDPGS KFHVPANVPYVRYFVSFIIQFQFHEALCRAAGHTGPLHKCDIYQSKEAGKLLADAMKLGYSKPWPEAMKL ITGQPNMSASAMMNYFKPLTEWLVTENRRHGETLGWPEYNWAPNTGTTPTLPPAPGPSS

>XP_694336.5 angiotensin-converting enzyme [Danio rerio] MNRGKRESRTPIGKQEHPAVNVSRRLLRKLLSQHSASQLRSIMLRAVVLLTLISWSAALKPEWMPGDYPP TEQGAERFVSDYNSTAEEVLYLSTEASWNYNTNLTDHNSQLQIEASLEEQAFTEAWGHKAKATFSESLMD TFTNPDLKKIIKKINVLEAANLAITDRELYNTILSQMDSIYSTAKVCPSPEECWSLEPELQEIMATSRSY KRLLYAWEGWHNSSGVPLKSLYAEFVKISNKASQMDGFKDTGEYWRSWYESPTFKQDLENLFKQLEPLYQ NLHAFVRRKLYDYYGPKYINLKGPIPAHLLGNMWSQTWNNIYNMMIPFPNRPNVDVTNTMIAKGYNATHM FRVAEEFFTSLGLLEMPPEFWDKSMLEKPTDGREVVCHASAWDFYNRKDFRIKQCTTVTMEQLFTVHHEM GHVEYYLQYKDQPVSFRRGANPGFHEAIGDVLSLSVSTPKHLHSIDLLDQLTDDAESDINYLLKMALEKI AFLPFGYLIDQWRWSVFSGETPPDRYNADWWYLRTKYQGICPPTRRTEEHFDAGGKYHIPGNTPYIRYFV SFILQFQFHEKLCKEAGHTGPLHKCDIYKSREAGAVLEKVLKAGSSEPWTQVLQEALGTDKMDATPLMSY FLPVTTWLREQNEKTGETLGWPDFNWVPPIPEGYPEDIGKITDEMQAKQFLDEYNSTAEEVWNAYTESSW TYNTDITEAHKDNMLQKNLEMANHTKIYGLEARKYDTSDFQDESVKRILSKLSDLERAALSAEDLVEYNN LLASMETLYSVATVCKDKSNCLPLDPDLNKIMAESRDYDELLFAWQGWRNASGREIRDSYKRYVELANSA AKSNGHTDNGAFWRSLYETPTFEQDLEALWKDLEPLYLSVHAYVRRALYKKYGPERINLKGPIPAHLLGN MWAQTWSGIMDLVNPYPDATQVDATPAMIAQGWTPKRMFEESDRFFTSLGLLPMPPEFWNKSMLEKPTDG REVVCHASAWDFYNRKDFRIKQCTVVTMDDLITVHHEMGHVQYFLQYKDQPISFRDGANPGFHEAIGDVL ALSVATPKHLQSIGLLDKVEDNAESTINFLMSIALDKIAFLPFGYLMDQWRWKVFDGRISSSEYNKEWWN LRMKYQGLCPPVPRTEKDFDPGAKFHIPANVPYIRYFVSFVIQFQFHKGLCDAAGHKGPLHNCDIYQSKE AGKLLSDVMKMGFSKPWPEAMKIITGQPKMSVQPLMEYFKPLIEWLEKENEKNGDVLGWPEYDWTPYKLS TVVEESPKSVNFLGLSVDAAGAAAGQWILLVLSIVFLLAVVFLAYRYSKTKRLQNKSMSQMELK

>XP_044035667.1 angiotensin-converting enzyme isoform X2 [Siniperca chuatsi] MRQQLQGKVFPWWRLAMGSGVDRFLWTVLLLLPVLGLSEALSESWQPGEYANNMTDALRFLSDYNRTAEE VFFFSISASWNYNTNITDHNSALQVNASLEEQAFSAAWGSKAKQVFSLAILNSLLDPKDKKLMKKIMILG AANLPQKEREEYNTILSTMDNIYSTTKVHPQPNISWSLEPDLTDIMANSRSYKRLLYVWEAWHNASGVPL KRYYPRFVELSNNASQADGFADTGADWRSEYESETFEQDIEKLYRTIEPLYQNLHAFVRRQLYNQYGPKY INLKGPIPAHLLGNMWAQTWNNIYGMMIPFPDKPNMDVTDEMGKQGYNATHMFRVAEEFFTSLGLEEMPQ EFWSGSMLVKPEGREVVCHASAWDFYNRKDFRIKQCTTVTMEQLFTVHHEMGHVQYYLQYKDQPVGYRRG ANPGFHEAIGDVLSLSVSTPKHLKTINLLENVTSDIETDTNYLLKMALEKIAFLPFGYLIDQWRWGVFSG HTPPERYNSDWWYLRTKYQGICPPTRRTEEHFDPGAKYHIPGNTPYIRYFVSFILQFQLHDKLCEAAKHE GPLHTCDIYRSKEAGAILKKILQAGSSKPWPDVLQEAIGTNKLDANSLIKYFDPIIKWLEKQNVNETLGW PDFSWVPPIPEGYPEDIDKNTDELDAKKFLDEYNSTAEGVWNAYTEASWKYNTDINEANKKAMLEKNLEM SAHTLKYGQKARQYDTTDFQEGSVKRIIKKLSDIERAALPSAELEEYNTLLSNMETKYSVAEVCRDNGTC HPLDPDLQKIIAESRDYDELLFAWKGWRDAAGKVLRQDYKRYVELANKAATLNGHSDNGAFWRSLYETPT FEEDLETLWKELEPLYQNVHAYVRRALYKKYGSKYINLKGPIPAHLLGNMWAQTWSGIMDLAMPYPHATQ VDATPAMVAQGWNATRMFQESDKFFTSLGLLPMPKEFWDKSMLEKPSQERQVVCHASAWDFYNRKDFRIK QCTVVTMDDLITVHHEMGHVQYFLQYKDQPVSFRDGANPGFHEAIGDVLALSVSTPKHLQSIGLLDKVEN NHEGDINFLMSMALDKIAFLPFGYLMDQWRWKVFDGRIPPTEYNKEWWNLRMKYQGLCSPVTRTEDDFDP GAKFHIPANVPYVRYFVSFIIQFQFHKALCDAAKHDGPLHTCDIYKSEEAGKLLGDVMKLGFSKPWPEAM AMITGQPKMSAQPLMQYFQPLIQWLEAENNKNNDIRGWPEYDWKPSSSSEIKANKVDFLGMSVDDSAAIA GQWVLLVVGLVLLVATIVLAYKYRSNKATMELKHN

>XP_043869570.1 angiotensin-converting enzyme [Solea senegalensis] MGTGVDRFLWSVLLLLPALGLCGALPADWLPGDYVDSVDDALRFLNDYNSTAEEVLFHSVTASWNYNTNI TDHNSMLQVNASLEEQAFSEAWGMKAKNTFTDATINKLTDPKDRTLMGKIKILGPANLPEEERKKYNTIL STMDSIYSTAKVHPQPNISWSLEPHLTNIMANSRSYKKLLYAWEGWHNASGVPLKKHYPGFVELSNKASK ADGFADTGADWRSWYDTPTFEADLEDLYKTIEPLYKNLHAFVRRQLYNQYGSKYINLNGPIPAHLLGNMW AQTWNNIYGLMIPFPEKPNLDVTDEMVKQGYNATHMFRVAEEFFTSLGLKEMPTEFWEESMLEKPEGREV VCHASAWDFYNRKDFRIKQCTTVTMEQLFTVHHEMGHIQYYLQYKDKPVGFRRGANPGFHEAIGDVLSLS VSTPKHLKTINLLESATSDPETDLNYLLKMALEKIAFLPFGYLIDQWRWGVFSGNTPPERYNSDWWYLRT KYQGICPPTRRTADHFDPGAKYHIPGNTPYIRYFVSFILQFQFHEKLCEAAKHTGALHTCDIYRSKEAGA ILQKVLQAGSSKAWPDVLQEAIGTRKMDAGSLMRYFAPIIEWLEKQNENETLGWPDFNWVPPIPEGYPED IDKNTDELDAKTFLEEYNSTAEEVWNAYTEASWKYNTDINDANKKEMLEKNLAMADHSLKYGQRARQYDT TDFQDASVKRIINKLSDIERAALPSAQLEEYNNLLSNMETKYSVAEVCREDGTCHPLDPDLQKIMAESRD YDELLFAWKGWRDAAGKEIRQDFKRYVELANQAAKLNGHSDNGAFWRSLYETPTFEEDLETLWKELEPLY QNVHAYVRRALYKKYGSKYINLKGPIPAHLLGNMWAQTWSGIMDLVMPYPDATQVDATPAMVAKGWNALK MFEESDKFFTSLGLIPMPSEFWEKSMLEKPSDGRKVVCHASAWDFYNRKDFRIKQCTVVTMDDLITVHHE MGHVQYFLQYKDQPVSFRDGANPGFHEAIGDVLALSVATPKHLQSIGLLDKVESNYESDINFLMNMALDK IAFLPFGYLMDQWRWKVFDGRIPPTEYSKEWWNLRMKYQGLCPPVTRTEDDFDPGAKFHIPANVPYVRYF VSFIVQFQFHKALCDAAKHTGPLHTCDIYQSKDAGKLLGDLMKLGFSKPWPEAMSMITGQPNMTAQPLME YFKPLIEWLEKENDNNKDVRGWPEYDWRPSADEVKIEEPKPTTVDFLGMNVNSSAAVAGQWVLLVIGLVL LLATILLAYKYRKSKKSKKSNSMMELK

>XP_039991037.1 angiotensin-converting enzyme isoform X2 [Xiphias gladius] MGSAVDRVLCSALLLLPAFGLSVALPESWLPGEYSNTTTDALRFLSDYNSTAEEALFHSVSATWNYNTNI TDYNSMLQVNASLEEQEFSEAWGLKAKHIFSNELLSSLPDPKDKELMERIMILGAANLPKKQREEYNTIL STMDNIYSTAKVHPQPNISWSLEPHLTDIMANSRSYKRLLYVWEGWHNASGVPLKKLYPRFVELSNKASQ ADGFADTGADWRSGYESETFEQDLEDLYRTIEPLYQNLHAFVRRQLYNQYGPKYINLKGPIPAHLLGNMW AQTWNNIYGMMIPFPDKPNLDVTDAMVKQGYNATHMFRVAEEFFTSLGLEEMPQEFWDESMLVKPDDREV VCHASAWDFYNRKDFRIKQCTTVTMEQLFTVHHEMGHVQYYLQYKDQPVGFRRGANPGFHEAIGDVLSLS VSTPKHLKTINLLESATSDTETDINYLLKMALEKIAFLPFGYLIDQWRWSVFSGRTPPERYNSDWWYLRT KYQGICPPTRRTEEHFDPGAKFHIPGNTPYIRYFVSFILQFQFHEKLCEAAKHTGPLHTCDIYRSTEAGL ILKKVLQAGSSKPWPEVLQETIGTNRMNASSLMKYFEPIIKWLEMQNKHETLGWPDFNWVPPIPDGYPED IEKNTDELDAKKFLDDYNSTAEAVWNAYTEASWKYNTDINEANKQAMLEKNLEMSAHTLKYGQKARQYDT TDFQDALVKRIIKKLSDIERAALPPAELEEYNTLLSNMETKYSVAEVCRDSGKCHPLDPDLQKIMAESKD YDELLFAWKGWRDAAGKVIRSDYKRYVELANKAATLNGHPDNGAFWRSLYETPTFEEDLETLWKELEPLY QNVHAYVRRALYKKYGPKYINLRGPIPAHLLGNMWAQTWSGIMDLVIPYPHATQVDATPAMVEQQWNATR MFEESDKFFTSLGLLPMPKEFWDKSMLEKPSDGRQVVCHASAWDFYNRKDFRIKQCTVVTMDDLITAHHE MGHVQYFLQYKDQPVSFRDGANPGFHEAIGDVLALSVSTPKHLQSIGLLDKVENNKESDINFLMSMALDK IAFLPFGYLMDQWRWKVFDGRIPPTEYNKEWWNLRMKYQGLCPPVARTEDDFDPGAKFHIPANVPYVRYF VSFIIQFQFHKALCDAAKHDGPLHTCDIYKSKEAGKLLGDAMKLGFSKPWPEAMTMITGQPKMSAQPLMQ YFQPLIQWLEAENNKNNDVRGWPEYNWKPSSMSSTFNEKQSIPRVTYSVFLCVCPDANRVNFLGMSVEGS AAIAGQWTLLVIGLVLLVATILLAYKYRKSRKPEKSLSTMELKQKD

>XP_041829354.1 angiotensin-converting enzyme isoform X1 [Melanotaenia boesemani] MGAGMNRLLSVLLLLPVLGFSEALRDSWLPGEYTNTTADAKRFLEDYNGTAEEVLFLSVSASWNYNTNLT EHNSKLQVNATLEEQAFSEAWGMKAKQTFSKEVLDSLADPKDKKLMEKIMSLGAANLPQVEREEYNTILS TMDSIYSTAKVYPQPNVSWSLEPELTDIMANSRSYKKLLFAWEGWHNASGVPLRKHYPRFVELSNKASQA DGFADTGADWRSWYETETFEEDIDKLFRTIEPLYLNLHAFVRRQLYNRYGPKYINLKGPIPAHLLGNMWA QTWNNIYDMMIPYPDKPNLDVTDKMVQQGYNATHMFSVAEEFFTSLGLKEMPTEFWEESMLVKPDDREVV CHASAWDFYNRKDFRIKQCTTVTMEQLFTVHHEMGHIQYYLQYKDQPVGFRRGANPGFHEAVGDVMSLSV STPKHLHTINLLESLTSDAETDLNFLLKMALEKIAFLPFGYLIDLWRWSVFSGRTPPERYNAEWWALRTK YQGICPPTKRTEEHFDPGAKYHIPGNTPYIRYFVSFILQFQFHEKLCQAANHVGPLHTCDIYRSAAAGAI LEKVLQAGSSKPWPEVLHEAIGTNKMDASSLMKYFEPVIKWLEKENMNETLGWPDFNWVPPIPEGYPEDI DKNTDELVAKKFLEEYNSTAEVVWNAYTEASWNYNTDINEANKQAMLEKNLEMSAHTLKYGLQARQYDTT DFQDSSVKRIIKKLSDIERAALPSAELEEYNTLLSNMETKYSVAEVCRDNKQCHPLDPDLQKIMAESRDY DELLFAWKGWRDAAGKVLRQDYKRYVELANKAAVLNGHADNGAFWRSLYETPTFEEDLETLWKELEPLYL NVHAYVRRGLYKKYGPKYINLKGPIPAHLLGNMWAQTWSGIMDLVIPYPHATQVDATPAMVAKGWNSTMM FQESDRFFTSLGLLPMPQEFWDKAMLEKPSDGRQVVCHASAWDFYNRKDFRIKQCTVVTMDDLVTVHHEM GHVQYFLQYKDQPVSFRDGANPGFHEAIGDVLALSVSTPKHLQSIGLLDKVESNHESDINFLMSMALDKI AFLPFGYLMDQWRWKVFDGRIPPTEYNKEWWNLRMKYQGLCPPVIRTEDDFDPGAKFHIPANVPYVRYFV SFIIQFQFHKALCTAANHVGPLHTCDIYKSQEAGKLLGDVMKLGFSKPWPEAMAIITGQSKMSAQALMEY FEPLIQWLEAENNKNNEIRGWPEYDWKPRLLPRQKGHSGTQWHLVFATLLQLTVKSILPSSHFNDEILLK DPAGENEVDFLGMRVDSAAAIAGQWILLVLGLVFLVATSFFAYKYRKSKKPEKSLSTMELKQKD

>XP_041818495.1 angiotensin-converting enzyme [Chelmon rostratus] MGTSVDRSVWTVLLLLLPVLGLSEALKEEWMPGNFTSTTEEALRFLQEYNSTAELVLFQSVSASWNYNTN ITDHNSVLQVNAALDEQAFAFAWGSMAKQVFPAELVNTLPDRKDRTLMEKIKLLGAANLNQADRQLYNER LSQMDKIYSTAKVHPEPDVSWSLEPHLTEIMASSRNYKQLLFVWDEWHKAAGVPLKSLFKDFVEVSNRAS QADGFKDTGAEWRSWYETDTFVEDLEEIYRTIEPLYKNLHAFVRRKLYNLYGPKYINLKGPIPAHLLGNM WAQTWNNIYDMMIPYPNKTNMDVTDKMVKDGYDAQRMFRVAEVFFTSLGLDPMPQEFWDESMLVKPEGREVVCHASAWDFYNRKDFRIKQCTTVTMEQLFTVHHEMGHIQYYLQYKDQPVGFRRGANPGFHEAIGDVLSL SVSTPEHLKAINLLESVSSDQGTDLNYLLKMALEKIAFLPFGYLIDQWRWGVFSGHTPEERYNTDWWYLR TKYQGICPPIKRSLDSFDPGAKYHIPGNTPYIRYFVSFILQFQLHEKLCAATKQNVSLHRCDIYNSKEAG AILKKILQAGSSQPWPDVLKEAIGTDKLNASSLMKYFQPITDWLEKQNVDETLGWPEIDWVPPVPEGYPD DINKNTNELEAKTLLDEYNATAEVVWNNYTEASWKYNTDINDENEEAMLQRSLAMSAHTLEYGKRAREFD TTDFQDSSVKRIIKKLSDIERAALNETDLKEYNTLLSQMETTYSVAKVCEGDKCYQLDPDLQKIMAESRD YDKLLFAWKGWRDSAGKALREQYTKYVELANRAARLNGHSDNGAYWRSLYETPTFEEDLEALWKQMEPFY QNLHAYVRRALYKKYGPEHIDLKGPIPAHLLGNMWAQTWSGIMDLAIPYPHATQVDATPAMVAQGWNATK MFHVSDEFFTSLGLLPMPQEFWDKSMLEKPSDGRQVVCHASAWDFYNRKDFRIKQCTVVTMDDLITVHHE MGHVQYFLQYKDQPVSFRDGANPGFHEAIGDVLALSVSTPKHLKSIGLLDKVEDNTESDINFLMSMALDK IAFLPFGYLMDQWRWRVFDGRIPTTEYNKEWWNLRMKYQGLCPPVPRSEEDFDAGAKFHIPANVPYVRYF VSFIIQFQFHKALCNAAGHEGPLYKCDIYNSKAAGKLLGDMMKLGFSKPWPEAMTLITGQSKMSAEPLKE YFKPLTDWLEKENNKNKEVLGWPNYDWTPSSTGVIEDNKVDFLGMSLDGSAATAGQWTLLALGLVFLLTT ILLAYKYRKSKKHHKSSSTMELK

>XP_030195515.1 angiotensin-converting enzyme [Gadus morhua] MDSFLWAAVLLLPLLGFSEAVPADWLPGPYTQTPEEAVRFASDYDLTAEKALFASVTASWTYNTNLTAHN SQLQVQASLDEQAFTEAWGNRAKQTFPPDFLATLTNNSTKKLIEKISELGPANLAEADRREYNTILSTMD DIYSTAKVCPDKDKPTECWSLEPELSDIMANSRSYKRLLYAWEGWHNASGVPLKKHYPRFVELSNKAVAP DGFADTGENWRSWYETETFETDLEELYKTVEPLYQQLHAFVRRKLHNQYGSKYINLKGPIPAHLLGNMWS QTWNNVYGLMVPYPEKPNVDVTDTMVAQGYNATQMFKVAEEFFTSLGLIKMPEEFWNESMLVKPEGREVV CHASAWDFYNRKDFRIKQCTTVTMEQLFTVHHEMGHVEYYLQYKDLPVGYRRGANPGFHEAIGDVLSLSV STPKHLHKIGLLDKVTDDPETDINYLLKMALEKMAFLPFGYLIDQWRWNVFSGKTTPDHYNADWWHLRTK YQGICPPTPRTEEHFDAGAKYHIPGNTPYIRYFVSFILQFQFHEKLCAAAKHTGPLHKCDIYQSKEAGAI LEKVLKAGSSKPWPEVLQDALGTNKMDAGALMKYFGPIITWLEEQNVNETIGWPDFNWVPPIPEGYPASI DKNTDELQAKQLLKEYNSTAERVWNTYTEASWAYNTDITEQHKDAMLAASLEMSKHTLEYGLKARQYDTS DFQDGAVKRIMNKLSDIERAGLPAPELEEYTNLLSNMETKYSVAEVCREDGPCLPLDPDLQKIMAESRDY DELLFAWKGWRDSAGKGLRKDFERYVELANTAARLNGHSDNGAFWRSLYETPTFEEDLETLWKQLEPLYQ NIHAYVRRSLYKKYGDKHINLKGPIPAHLLGNMWAQTWSGIMDLTIPYPDATQVDATPAMLAKNWNATMM FQESDRFFTSLGLLPMPKEFWEKSMLLKPTDGKKVVCHASAWDFYNRKDFRIKQCTVVTMDDLITVHHEM GHVQYFLQYQDQPVSFRDGANPGFHEAIGDVLALSVATPKHLKTIGLLDKVENNHDSDINFLMSIALDKI AFLPFGYLMDQWRWKVFDGRIPASEYNKEWWNLRLKYQGLAPPVARTEEDFDPGAKFHIPANVPYVRYFV SFIIQFQFHEALCKAANHQGPLHTCDIYQSKEAGKLMGDVMKLGFSKPWPEAMAMITGQPRMTAGPLIEY FKPLIKWLEDENAKNKEVLGWPEYAWIPPSEPVVVVPEVVVPEVQGVNFLGMSLDSAAASAGSWVLLVIG LVLLVATILFAYKYRKSRKISKSEGMSMH
