# Supplementary material for: The role of the renin-angiotensin system (RAS) in salinity adaptation in Pacific white shrimp (Litopenaeus vannamei)
Source: Front Endocrinol (Lausanne). 2022 Dec 15;13:1089419. doi: 10.3389/fendo.2022.1089419 (PMC9798321; doi:10.3389/fendo.2022.1089419)
Supplement: Supplementary file 3 [file DataSheet_3.docx]

**The sequences of the amplified products for LV-APN gene**

>LV-APN (Present study)

TTTTTTTCTTCTCTCGCGACTGCAGCCCCTCGCACAGTTAGCTCCCGACAACGGATCGCACCGCACTGTGCCCGGAGAAGTTTCGGTCCCGGGGTGAGCAGCGTAGAAGACGGTGGCGAGATATTTCATTTATCTTTATTCTACTGGTCTGATTGTTGGGATCGATTCCTCTGGTTATAAATAGCATACAGGAGTGAGGAGGTTCTGCAACAGCCAATGTTCCTTGGAGTCTCTTCTGCCTCAGGCTGTAAGAATGCTGCTGTTTGTTCTGCTCGCCCTCGGTCACGCCACCCAGGGTTTACCTAATGTTGATGACGTCACCATTCCACCTTGGGTGACCACTACAACTGAGGCATCTAGATCATTGGGACCTGTAGACTTGGACGTACAACTACCTCGATCCATTAAGCCGCTCCATTATGTGGTCAAACTTCAACCCTTCATCAATGGCAACTTCAGCATCGTCGGCTACATGGAGGTGGAGATGGAGGTTCTGGAACCCACTTCCAACATCACTCTCCACATATCGGATATCATCACTAAAAACGACACCGTAAAGGTTTCAGATCAAGCAACATCCCGAGGTTTGAGGATCAAGAAACACGAGTATGACCATAGTCGTCACTTTTACATTGCCCATTTGAGGAAAGAGCTCCAGAAGGGGAAAAGGTACATACTGTCCATGGAGTTCCTTGGTTATCTTAATGATAAGCTGCGTGGCTTCTACAGAGCGACCTACAAGGATGTTGATGGTAACATCAGAAATGCGGCTGCGACCCAGTTCCAGCCCACCGACGCCCGCAAAGCCTTCCCTTGCTTCGACGAGCCCGCACTGAAGGCAACCTTTGAAATTCACCTCGCAAGGGAATCTTGGATGACGACCCTCTCCAACATGCCCATTGCCGAAACGGTGCCTGTTGAAGGACAGGAGGGATGGATGTGGGATCGTTACGAGAAGAGTGTCCCTATGTCCACTTACCTGGTTGCCTTCGTTGTGTCCGACTACGTTCATATCAACTCGACCGAGAATGACCGTGTAGATTTTCGAGTGTGGGCACGACAAGAGACCATAGATCAAGCAGAGTATGCAAATGAGATAGGTCCCAAGATCTTACGTTTCTTTGAAGATTATTTCAACCTATCTTATCCCCTTCCGAAGATGGACATGATTGCATTAACCGACTTCTCTGCAGGAGCTATGGAGAACTGGGGTCTAATAACTTACAGAGAAAGTATCTTCTTGTATGACCCACAAGTTTCAACCCCAGTCGACAAGGCAGTTATTGCAAGTATTGTATCTCATGAGTTGGCTCACCAGTGGTTTGGCAATCTGGTGACGCCTAAGTGGTGGGACGACTTGTGGCTCAACGAGGGATTCGCTACTTATATAAGTTACCTTGGTGTAGATCATGTAGAACCAACGTGGAAGGCAATGGAAGAAATCGTCGTTGAAATAGTCCATCGTGTGTTCGATCTAGACAGCCTGGAGTCCTCCCACAGGATCAACATTCCCGTCTCAAACCCGGACGAAATATTCGAAGTCTTTGATGACATATCTTACAATAAAGGAGCGTCTATCATCCGGATGATGACCCACTACCTCACAGAGCCGACCTTTAGAAAGGGATTGAACAACTATCTAAAAGCACTCACATACAATAGTTCTGTGCAAGACGACCTGTGGAAGCATCTGACTCTGGCGGCTCACGAGGACGGCATTCTACCCCAGGAGGTGACTGTGAAGATGATCATGGACACGTGGACGCTGCAGATGGGATACCCCGTCGTGAAGGTGACAAGGAGCCCCGACGGAACCTCTGCTACTTTGACACAGGAGCGGTTCCTCTTAGAAGGGAGCGCAAACTCTTCCAGCACCACGGATTACAAGTGGTGGGTGCCGCTGACCTTCACGACCCAGAACGAGGCCAACTTCAGCCAGACTCAAGCCAGTTTATGGATGAAGGACTCTGAGGATCATGTCACCGTCTCTTCTCTTCCCCCGAAGGACCAGTGGGTCATCTTCAACCTGCAGCAGGCGGCCTACTACCGAGTCAACTACGACGACCACAACTGGAATCTTATTATCCAACAGCTGAAGAAGGACCACCAGGTTATTTCTCCTGTCAACAGAGGGCAGATTGTCGATGATGCCATGAACCTTGCTAGGGCTGGTCATCTCAGCTACAAGCTCGCCCTGGACGTTTTACCGTATCTGCGAAATGAAGGAGAATATCTGCCTTGGGCTACAGGCGCCGATAAATTCGTTTACATAGAGAGTATGTTCAAACGCAGAAGTGGATTCGGCGCTCTCAAGCGTTACCTCCTGGATTTGGTGCTGCCGCTCTACGAGTCCGTTGGGTTCGACAACAACATCGAGGACTCTTTCTTGGAGCAACAAAGGAGGAAAATAGCCGTGAAATGGGCTTGCAGACTGGGCCACAAGGACTGCCTCAACAAAGCGCTCACTCTCTACAGGCACTGGATGTCACAGCCGGACAATTCGAGCATCATTTCGCCAAACCTTAAACCTGTGGTGTATTGTCGCGCCATCGCCGAGGGCGGGGAGGCCGAGTGGGACTTCGCGTGGGACCAGTACGTTACTTCCAGCGTCGCTACCGAGAAAAAGCTTCTTCTCAGATCTATGTCCTGCTCGAAGCAGACTTGGATCCTATCTAGATACCTCGAGATGGCTATGAACAGGAGCAGTGGCATAAGACTGCAAGATGTTATTTATGTGTTAAGAAGTTTGAGTAACAATGACGTGGGGCGCTCCATGCTGTGGGACTACCTCACACATAACTGGAACAATATTTATACATACAAGAAGAGTCGGAGAGGTGACGTCATGAAAAGGGTAACACAAGCATTTAATACGAAACAGGAACTGAAAAGGGTCCAGTCGTTTTTGAGGAGGAGGACAGTGACCTTGGAGGGGAACCAGAGGAGCGTCCAGCAGGCAGAAGAGAAGGTCAAGAACAACGTCGCCTGGATGGACACAAAGTATGACGTCATAGTCCAGTGGCTGGAGGAGAACGGCTACTCCTCGAAACTACGGGTCGTTTAATTCCATATATATACATACACACACACACACACACAC

**The protein sequences that used for bio informatics**

**>Present study aminopeptidase N-like [Penaeus vannamei]**

MLLFVLLALGHATQGLPNVDDVTIPPWVTTTTEASRSLGPVDLDVQLPRSIKPLHYVVKLQPFINGNFSIVGYMEVEMEVLEPTSNITLHISDIITKNDTVKVSDQATSRGLRIKKHEYDHSRHFYIAHLRKELQKGKRYILSMEFLGYLNDKLRGFYRATYKDVDGNIRNAAATQFQPTDARKAFPCFDEPALKATFEIHLARESWMTTLSNMPIAETVPVEGQEGWMWDRYEKSVPMSTYLVAFVVSDYVHINSTENDRVDFRVWARQETIDQAEYANEIGPKILRFFEDYFNLSYPLPKMDMIALTDFSAGAMENWGLITYRESIFLYDPQVSTPVDKAVIASIVSHELAHQWFGNLVTPKWWDDLWLNEGFATYISYLGVDHVEPTWKAMEEIVVEIVHRVFDLDSLESSHRINIPVSNPDEIFEVFDDISYNKGASIIRMMTHYLTEPTFRKGLNNYLKALTYNSSVQDDLWKHLTLAAHEDGILPQEVTVKMIMDTWTLQMGYPVVKVTRSPDGTSATLTQERFLLEGSANSSSTTDYKWWVPLTFTTQNEANFSQTQASLWMKDSEDHVTVSSLPPKDQWVIFNLQQAAYYRVNYDDHNWNLIIQQLKKDHQVISPVNRGQIVDDAMNLARAGHLSYKLALDVLPYLRNEGEYLPWATGADKFVYIESMFKRRSGFGALKRYLLDLVLPLYESVGFDNNIEDSFLEQQRRKIAVKWACRLGHKDCLNKALTLYRHWMSQPDNSSIISPNLKPVVYCRAIAEGGEAEWDFAWDQYVTSSVATEKKLLLRSMSCSKQTWILSRYLEMAMNRSSGIRLQDVIYVLRSLSNNDVGRSMLWDYLTHNWNNIYTYKKSRRGDVMKRVTQAFNTKQELKRVQSFLRRRTVTLEGNQRSVQQAEEKVKNNVAWMDTKYDVIVQWLEENGYSSKLRVV

>XP_047472222.1 aminopeptidase N-like [Penaeus chinensis]

MLLFALLVLSHTTLGLPNADDVTIPPWVSTESHPTTTEASTSSLFTTKKAENFDVRLPRSLKPLHYLVKL QPFINGNFSIIGYMEVEMEVLEPTSNITLHISDIITKNDTVKVYTSGQAQRRGLRIKKHEYDRGRQFYIA HLRKELQKGKKYILSMEFLGYLNGEMHGFYRATYKDVAGNTRNVAATQFQPTAARKAFPCFDEPALKATF EIHLARESWMTTLSNMPIADTVPVEGQEGWVWDRYEMSVPMSTYLVAFIVSDYVQINSTVNDSVAFRVWA RQETIDQAEYANEVGPKILGFFEDYFNLSYPLPKMDMIALTDFSAGAMENWGLITYRESFLLYDAQVTTP VDKSIIAEIISHELAHQWFGNLVTPKWWDDLWLNEGFATYISYLGVDHVEPTWKVMETTLVEIVHNVFGL DSLESSHRISIPVSDPDEIFEVFDDISYSKGSSIIRMMTHYLTEATFRKGLNNYLKALTYNSSVQDDLWK HLTVAAHEDGILPKDVTVKMIMDTWTLQMGYPVVKVTRSADGTSAILTQERFLLETSANSSNTTGYKWWV PLTYTTQSEPNFNQTQAKLWMKDSEDHVTVTSLPPKDQWVIFNLQETGYYRVNYDDHNWNLIIQQLKKDH QVFCPVNRGQIIDDAMNLARAGHLSYKIAIGAYAYLRKEDEYLPWATGVNKLYYIESMFKRRSGFGALKH YLLDLVLPLYESVGFDNKIEDSFLEHQKRKIAVKWACKLGHKVCLDKVLTLYRHWMSHPDNTSIISPNLK SAVYCHAIAEGGEAEWDFAWDQYITTSVATEKKLLLNSMSCTKQAWILSRYLEMAMNPGNGIRLQDAIYV LRSMSNNDVGRSLIWDYLTLHWNNIYTFKKRRRGEVLKRVTQIFNTKQELEKVQSFLRRRTVSLEGNQRN VQQAEEKVKNNVAWMDANYDVIVQWLEENGYSSKLRVI

>XP_042891875.1 aminopeptidase N-like [Penaeus japonicus]

MMLLVALLALSHATLGIPNVDDVTIPPWVSTERQPTTTEGTTTPSIITTKAAEKLDVRLPGSLKPLHYLV KLQPFINGNFSISGYAEVEMEVLEPTSNITLHISDIITKNDTIKVHPLGQARTRGVRIKKHEYDHARQFY IAHLKNPLQKGTKYVLSMEFIGYLNAELHGFYRSSYTDIDGSIRNVAVTQFQATYARKAFPCFDEPAMKA TFEIHLARESWMTTLSNMPIAETVPVEGQEGWVWDRYERSVPMSTYLVAFVVSDFAHVNSTEIEHVVLRV WARPEAIDQAEYANEVGPKILSFFEEYFNLSFPLPKLDMIAVPELSFGGMENWGLITYREKYLLHNPRVS TPLSKTFVAELISHELAHQWFGNLVTPVWWDDLWLNEGFATYISYLGVNQVEPAWKVMETTLVQTVHKVF GLDSLKSSHRISIPVDHPDEISEVFDAISYNKGASIIRMMVHFLSEPTFRKGLNNYLKAFTYKNAVQDDL WQHLTVAAHEDGVLPQDVTVKMIMDTWTLQKGYPVIQVMRSVDGTSAILAQDRFLLERGANSSNTTDHKW WVPLTYTSQSEADFNRTQAALWMKASEDHLTVSSLPPKDQWVIFNLQETGYYRVNYDDHNWNLLIRQLKD NHEVICPVNRGQVIDDAMNLARAGHLNYKVAIGVFSYLRNEGEYLPWSTGVDQLDYIESMFKRRSGFGAL KRYIRDLVLPLYRAVGFDARAEDSFLEQEKRKIAVNWACKLGHKDCLDEVLTRYRQWMSQPDNTSIISPN LKSTVYCRAIAEGGEAEWDFAWDQYLRTNVASEKTLLLSAMACSKEAWILSRYLEMASNPGNSIRQQDII IILRSLSANDVGRPLVWDYLTLRWNDIYIYKKRRRGDVLKRVTQTFNTKQELELVRSFLRRRRVSLEGNR RSVQQVEEKIKNNVAWMDANYDVIVQWLEENGYSSKLRVV

>XP_045118108.1 aminopeptidase N-like [Portunus trituberculatus]

MKKSKLGAAPEEGGGGGGGGGGGGGGGGEGGGDLLTMEGSNHHHHHHSTPPSCTKNKGCFVGRGVGVLLG VMFVSGLVATGLLVYYYAPHEKQMQEEAVLVPRVQSNVRRTPNTRRRLNCTARRFGGSRGAATTTSTTFP SSTLISIPSLFSTFVPFEEEPDVRLPRTLRPLHYLVKLQPLINGNLSILGYMEVEMEVLEATVNITLHIA DIITHNDTIRVTTPGDLDGPGLKIRKHEYDHERQFYMAHLEEPLVEGGKYVLSMGFEGHLNDQLHGFYRS TYRDADGTEKAMGVTQFQATDARRAFPCFDEPALKATFEVYLGRQMNMTTISNMPIIETIPMVNQKGWVW DHYDTTVPMSTYLVAFVVSDFANLSTIANNKTLFRVWARNSAIRQAEFAQQVGPPILLYFEDYFNQSYPL PKQDMIAIPDFSAGAMENWGLITYRETAMLYDARVSAASNKQYVAAVVAHELAHQWFGNLVTPRWWTDLW LNEGFASYVEYIGVNHVAPTWQVMEQFVLNELHIVFGLDSLESSHPISIPVGHPDEISQIFDRISYAKGA SIIRMMNHFLTESAFRHGLSTYLDAFKYDNAEQDDLWRHLTEAAHETGTLPANLTVKTIMDTWTLQMGYP VIKVERSADGTSAAVSQERFLLVKDDNADDMHDYKWWVPLTYTGQDRPDFSQTQAKVWMKDSEAQVTVTS LPSKDHWVIFNLQQTGYYRVNYDDHNWNLLIQQLLADHRAIATVNRAQIIDDAMNLAKAGQMTYGTALGV YNYLGAETEFVPWAAAIDNLAYLKEMFARTGGYGALKNYLLDILVPLYDSVGFQDRDNDPLLDQYKRQKA LSWACQLEHPDCLQNAAASYATWMADPSNNSIISPNLKSTVYCHAIGSGGEKEWNFAWEQYLASNVGSEK NRLLSAMGCTKHIWILSRYLEMAFSSDSGIRKQDAFRVFGTVANNDVGQPLAWSFLQDRWHDIYEFYGKA KSRLIKSATRSFNTHLQLKELRLFHREHKEDLGSASRAVQQVIERTKVKIAWMRTNYDVIVEWLDANGYS TKLRNA

>XP_045610140.1 aminopeptidase N-like isoform X1 [Procambarus clarkii]

MKAADGSKPAASVNRDGCHVRRGAALVLGFLVVSTAVAADSFVPLRSSHHKLASVEAQGSEEVDIRLPTA LKPLHYIVKLQPFINGNFSIMGYVEIEMEVLEPTTNITLHIVDIITKNETIKVVPSDDVNGTGIEVQQNL YDNDRQFYTGVLGEELEVGRNYILSMQFLGYLNDELRGFYRSTYKDQDGNTRFMASTQFESTDARRAFPC FDEPAMKASFEIFLAREASMSSISNMPKYETFPVEGQEGWEWDHFNTSVPMSTYLVAFVVSDFGHVDSNA NDHVLFRVWVREDAIQQADYAINTGPEVLTFYEDYFNIPFPLPKQDMIAIPDYSGGAMENWGLITYRESA LMFDPEASSAYSKQYVTVIIAHELAHQWFGNLVTPDWWTDLWLNEGFASFMENIGTNHIDPTWRMWEQFV TDYLQMVLELDSLESSHPISVPVGRPEEIDQIFDDISYSKGSSVIRMMNDFLTETTFRKGITNYLNGQAY SNADQDDLWRYLTEAAHEDGSLPSDTTVKMIMDTWTLQMGYPVIKVVRSADGTSATLTQERFLLVKSANS SDTHDYKWWVPLTYTSQDDPNFNNTQAMVWMKDSDPEITISSLPGKDQWVIFNIQETGYYRVNYDEDNWN LLIQQLNTDHQVIHVINRAQIIDDAMDLAHAGQLSYNTALSVNGYLKAETEYTAWRTAINNLDYLEMMFT RTGGYGALRRYLLSLLTPLYNAVGFDDNRSDPQLDQYKRVLALTGACSLGYQDCVDNSVSLFQTWMLNPS NTSIVSANLKSTVYCTAIAAGGEEEWNFGWNQYLASNLGSEKSTLLSALGCTKQIWILSRYLDMAFTADS GVRSQDASTVFSSVSRNDIGRDLAWNYLRDQWQHISNYVSSFTTLSDLVEAATAEFNTNEEKHELEMFKE EHPEDLHTAAQAVDQAIERTANNIAWMSNNYDVIVQWLDDQGFSSQLARRPPPP

>XP_037798518.1 aminopeptidase N-like isoform X1 [Penaeus monodon]

MLLFALLVLSHATLGLPNADDVTIPPWVSTESQPTTTEASTSSLITTKKAENFDVRLSRALKPLHYLVKL QPFINGNFSIIGYMEVEMEVLEPTSNITLHISDIVTKNDTIRVYTSNQAQRRGLRIKKHEYDHGRQFYIA HLRKELQKGKTYILSMEFLGYLNDEMHGFYRATYKDVDGNTRHVAATQFQPTAARKAFPCFDEPALKATF EIHLARESGMTTLSNMPIAETVPVEGQEGWVWDRYEKSVPMSTYLVAFIVSDYVQINSTVNDRIAFRVWA RQETIDQAEYANEVGPKILSFFEDYFNVSYPLPKMDMIALTDFSAGAMENWGLITYRESFLLYDAQVSTP VDKSITAEIVSHELAHQWFGNLVTPKWWDDLWLNEGFATYISYLGVDHVEPSWKTMETTLVEIVHNVFGL DSLESSHRISIPVSDPDEIFEVFDDISYNKGSSIIRMMTHYLTEATFRKGLNNYLKALTYNSSVQDDLWM HLTMAAHEDGILPQNVTVKMIMDTWTLQMGYPVIQVTRSPDGTSAILTQERFLLERSANSSNTTDYKWWV PLTYTTQSEANFNQTQASLWMKDSEDHITVSSLPPKDQWVIFNLQETGYYRVNYDDHNWNLIIQQLKKDH QVICPVNRGQIIDDAMNLARAGHLSYKIAIDVYAYLRKESEYLPWATGVNKLCYIESMFKRRSGFGALKR YLLDLVLPLYESVGFDNKIEDSFLEQQKRKIAVTWACKLGHKDCLDKVLTLYRHWMSHPDNTSIISPNLK ATVCCHAIAEGGEAEWDFAWDQYITTSVATEKKLLLHSMSCTKQAWILSRYLEMAMNSGNGIRLQDAIYV LRAMSNNDVGRSLIWDYLTLNWNNIYTFKKRRRGDVMKRVTQTFNTKQELEKVQSFLRRRTVSLEGNQRN VQQAEEKVKNNVAWMDANYDVIVQWLEENGYSSKLRVV

>XP_042215808.1 aminopeptidase N-like [Homarus americanus]

MLAGKRDGGGGGAPGSCCLLLLLLVTGPLYTSAFRRLRDELPTLLPLNTLEGQDNQEHFMTTLTSTQQHT TGPKTPVPTTPIEVSQEMSAEEPGPDTPLPRSVRPLHYLVRLQPFLNGNFSVHGFVEVEVEALESTTNIT LHLADLRVIVNSVKLVAVGEVEDGHSAPTITHFTGHSGVHTFTAHLTKPLNPGRRYRYSMNFYSNLKNTD KGFYSVKYKDEDGGERSLAITKFPPSYARRAFPCFDEPHLKATFDIQLARENNMTSVSLMPLVDTSPLEG QEGWVVDSFLTTPHIPTHSLAFAVSSLAPARNTSLSGLPITVWARQGVLQHTTFLQEFTPRFLTFFEQYF NVSYPLTKLDVVIVPGNETQAISSPGLIVIYQEAAGMYDASSSTSSHKQHVAEVLAHEVSHQWLGNLVTP KTWSHIWLYEGLASYLDNVAVSSVESSWSLNNDQVKDLQKALHKDSLSSSFPLYMPALNSEDIVDFLLNL VFQKGSSVVRMLRHCLSEKTFRTGLTNFIQRHKYDTAEQDDLWRHLTTAAHQDDTLPQDLTVKTIMDTWT LQMGYPVIKVERSPDGTSATVSQESFRLSQSEASNKWWVPLTYTVGDDPNFNETRAKVWMKDSETHITIP SLPTKDQWVIFNLQETGYYRVNYDQDNWNLLIQQLLTDHRVINTINRAQIIDDAMNLAGAGKLSYETALN VTKYLTKEKDAKVWDVVLTSLDHLNTMLANTAAYGGFKKYWLSVITPVYEKLSAAGKVPNSPWCYEVVKW ACYFGHPRCVQDSQTLYRQFMEKPNATSLAPEGMLEVVYCTAIAHGGEEEWQFGWTQYLTSDDDHQKDQL LKGLACTKESWMKNRYLDMAFTPGVNIRNHDTFQVFSSVAAQVEGPPLAWEFILQNWENVTAMFGTEDSL VDVIVRTASKPFNTQYQLAELEVFLEEHQDQPSVVEASRKAIQQTANNVVWMDTNYHVISQWLHERGFST RLQTL

>XP_043652209.1 aminopeptidase N-like isoform X1 [Drosophila teissieri]

MTYTPVSTMKWFLWVVLVISLALCSANSISSYNHYRLPTALRPQKYYLRILTLLENPDDLRFAGSVQIVI EALENTRNITLHSKNLTIDESQITLRHISGAGSKDNCVSSTSVNPTHDYYILHTCRELLAGNVYTLCLPF SADLSRQLHGYYRSSYKDPVTNTTRWLSATQFEPAAARKAFPCFDEPGFKASFVVTLGYHKQFTGLSNMP VKEIKPHESLPNYVWCEFEQSVPMSTYLVAYSVNDFSFKPSTLPNGALFRTWARPNAIDQCDYAAQFGPK VLQYYEQFFGIKFPLPKIDQIALPDFSAGAMENWGLVTYRETTLLYSPTHSSLSDQQNLANVIAHELAHQ WFGNLVTMKWWTDLWLNEGFATYVAGLGVQEIHPEWHSRDKGILTALITSFRLDSLVSSHPISRPIQMVT EIEESFDAISYQKGSAVLRMMHLFMGEESFRSGLKEYLQLYAYKNAEQDNLWQSLTSAAHQSGALAEHLY IKTIMDSWTLQTGYPVLNITRDYAAGSAKLTQERYLRNSQIPRAERVGCWWVPLSYTTQVEKHFNNTAPR AWMECSKTGESVPTTIDLLPGPEEWVIFNIQLSTPYKANYDARNWKLLIATLNSEEFQSIHVINRAQLID DVLYFAWTGEQDYDTALQVTNYLQRERDLIPWKAALDNLKLLNRLLRQTPNFGLFKRYMKKLLTPIYEHL NGINDTFSSITQQDHVLLKTMVVNVACQYQVGDCVPKALAYYRNWRSEANPDESNPVPINLRSTVYCTAI AQGTEEDWDFLWSRFKKSNVGSEQQTILSSLGCSKEVRILQRFLERAFDPEGAIRKQDSLLSFQAVVSGE QGFPLAKNYLMENVDFMVAYYYPQTRSMARLLPPLCEQIATTNDLNEFRAFVNKSQQSLKGVQQAVQQSL ETMVTNVQWMERNYPQILSSLQRNL

>XP_035893399.1 aminopeptidase N-like [Anopheles stephensi]

MTVMRSALLLVAILAAIAGTLGDSSNSYSSYRLPKSITPEHYNLRVYTHLGDERGFIFYGRVAIRFLCHE AADSVVLHSKNLTLLEQQITLHEVSPDVPQKASREMDVKGVEYITEHDYAVFHVSTALRKGARYELTIPF ESGLGTGLLGYYRSSYLDKRTKQKVWLAVTQFEPTYARQAFPCFDEPEMKATFDISLAHHERYVALSNMP VNRTVPVEDMPGWVLDEFNTTVPMSTYLVAYTVNDFEYREATTSEPGDVVFKIWARRDAIDQVDYARDIG PRVTRFYEDYFHQKFPLPKIDMIAIPDFASGAMENWGLITYRETALLYHPNISTASNKHRVASVIAHELA HQWFGNLVTMRWWTDLWLNEGFATYVASLGVDYLHPEWYSLEEESISNTLDIFKFDALLSSHPISVEIGH PNQISQIFDAISYEKGSIVIRMMHLFLSEETFRDGVSRYLRRHAYGNAEQDNLWEALTEEAHANGVLPDF IDVKRVMDSWTLQTGYPIITVTRNYDANSAEITQTRFVSSEVPADRNVTDYCWWIPLTYTTAKSLDFNDT LPKGWMECTNGTSNGHQVKVMDDLPDSEHWVIFNVQLAGLYKVKYDKTNYRLIIAQLNGPSYDAIGLLNR AQLIDDAMDLAWTGQQNYGIAFAMINYLRQETEYIPWKSALTNLNSLNRILKRTPLYDIFKSYVQYILEP IYERLEVFNVTRKPTDRLDAIKQLTLIASWACRFEVGDCVDRSVQLFARWMNEANPDANNPVPIDLRPVV YCNAIRQGNDTQWNFLWRRYLQSNVGAEKIMIIGSLACTRQLWLVERFLQWSLNSTSGVRKQDATILFSS VSRNDAGFNAAKSFFLTRADEIYEYLSPDTSRLSRYIKPLAEQMFSTQEVQELNELIRKKTALFEKANQG VKQALETAQTNSKWAETNINKMERLLPMLTSRSAADHLLNLIDT

>XP_040229772.1 aminopeptidase N-like isoform X2 [Anopheles coluzzii]

MAMGRSSMLLAVIAATIIGVTVGDTGVPYSGYRLPKSITPEHYNLRVYTHLGDERGFIFYGQVAMRLICH EDTENIVLHSKNLTLPEQGISLRELGSAGQQNGSAIAIKSVQYAKEHDFVILNVATVLRKGNRYELVVPF ESALGTGLLGYYRSSYVDKASKQKIWLAVTQFEPTYARQAFPCFDEPEMKATFDIALAHDERYVALSNMP VNSSAPVDGMPGWVMDVFGTTVPMSTYLVAYTVNDFEYREAMAAEEGDVLFKIWARRDAIEQVDYAREIG PKVTRFYEDYFQQKFPLPKIDMIAIPDFASGAMENWGLITYRETALLYHPNVSTASNKHRVASVIAHELA HQWFGNLVTMRWWTDLWLNEGFATYVASLGVDYLHPEWHSLEEESVSNTLDIFKFDALQSSHPISVEIGH PNQISQIFDAISYEKGSIVIRMMHLFLDEETFRDGVGRYLRRHAYGNAEQDNLWAALTEEAHANGVLPDH IDVKKVMDSWTLQTGYPIITVTRNYDANTAEVTQMRFISSDVRPDSNVTDYCWWIPLTYTTAKQIDFNDT LPKAWMACSGEPKGSHQQEAKLLEDLPDGDQWVIFNVELAGLYKVRYDRRNYQLIIAQLNGPRFGEIGLL NRAQLIDDAMDLAWTGQQNYGIAFAMLNYLRQETQYIPWKSALTNLNNINRILKRTPLYGVFRNYVQYIL EPIYEQLDIFNGSRATTERLDGIKQITLIASWACRFEVGDCVNRSVELFARWMNESSPDTDNPVPVNLRP VVYCNAIRRGDEAQWHFLWLRYLQSNVGAEKIMIIGSLACTREVRLVERFLQWSLNSTSGVRKQDATILF SGVSRNDAGFAAAKKFFLERADDIYNYLSPDTSRLSRYIKPLAEQMFSSEELQELTDLIEQKAPIFEKAN QGVKQALETAQTNNRWSKVNIDKMERLLPMLTTRSVSVLSLIDDL

>XP_032523235.1 aminopeptidase N-like isoform X1 [Danaus plexippus plexippus]

MECLKVLFLLSSVQLSRQYLLPDHIAPSHYQLRLLYDIDPSTNFSFFGVADIQLTVKKSTSKIILHAQDY MISDDKVSVVGQKEVPKVTGVKLNDTYNFLEISLDKDLEENGKYKLTIPFYGNLVKGLDGAYISSYTNRQ TQKTEYLISTQFEAISARKGFPCFDEPMYKATYSIIIGHSKEYTAVSNMPLAASASENALEDYWPWDVVG KRFRKERSSFVWDQFAKSVPMSTYLVAFVVSKFSHVVSPPELSKTQFRIWARGDAIDQTSYAAKIGPQVL SYFEKWFNVSFPLPKQDMMAIPDFSAGAMENWGLITYRETALLYSDKESSFLNKERIAEVVAHELAHQWF GNLVTMKWWSDLWLNEGFATFVSSVGVSAVEPTWRADRSYAVENTLSVLSLDALESSHPVSAPLDDPKRI SEIFDAISYRKGSTLIRMMLMFLGEGVFRQALHNYLMKYSYSNAEQDDLWAELTAASLRSGSLTRNITVK EVMDTWTTQTGYPILTVTRDYSDKSLTISQKRYLSLGVGRTSQAWWVPLSVLCEKDRKSESESVQWLGDT EGVTNEHRYEHGSGASEWVLFNYNMIAPYRVNYDQRNWKLLIQTLTSDQYTLIPVEGRVQLLSDAFELAW NNQLDYGMTLQLASYLKRETEYLPLYTGLSALAKIENVLKRSSEYGAFQKFIRRLLNNVYQKGGLALKRI VDGDDLNSVKLQTTVSSWACSVKIPGCEENAIDMFNDWMRTDRPDENNPIPVDLRRTVYCSAIRRGGVSL WRWSLARRRASNVATSRDALQHALACSRDVWVLAQYLEWTVSDGSEVRRQDAGNVIAAVTRSATGYYVAK DFIYGRIQEISKAFNGQDRRMGGIIKTLLGQFTTKKELDEFLEWKKLNEKYLSASKIAVAQGIENARVNI EWIQRNKRTVVDKMREYSIDFIDYEDKVVLRSFSVQVKSVTCAVLHSVVISVLFV

>XP_019964817.1 PREDICTED: aminopeptidase N-like isoform X2 [Paralichthys olivaceus]

MKDHSYHLYTNFTGELADDLGGFYRSEYMENGERKVVATTQMQPTDARKAFPCFDEPAMKAVFYITLIHD HGTVALSNGAEKESSSVNIHGKNLQQTVFEPTEKMSTYLLAFIVSEFTFINNTVDDVSIRIFARKPAIDA GQGAYALSKTGPILKFFEKYYNSSYPLPKSDQIALPDFNAGAMENWGLITYRETALLYDETFSSNSNKER IATIISHELAHMWFGNLVTLRWWNDLWLNEGFASYVEYLGANEAEPDWNIKDLIVLNDVHRVFAVDALAS SHPLSSKEDDIQRPEQISELFDAISYSKGASVLRMLSDFLTEEIFVMGLRTYLKKFAFGNAVYTDLWEHL QMAVNASGTDLPGSVHDIMNTWVLQMGFPVVTINTTTGDISQKHFLLDPDSNVTALSPFNYEWIVPIKWT KTGAPQVPYWLTQKSATNESMKTTGSDWVLANINVVGYYRVNYDDSNWDKLLNVLSTNHSLIEVINRAQL VDDAFNLARAKIIPTVRALSTTRYLNKETDYMPWDSALGNLNFFYLMFDRSEVYGPMQDYLRKQVVPLFN YYKNLTDNWSKVPTGHMDQYNQVNAISQACRTGHEECQTLVKGWFKQWMDTKKNMIHPNLRSTVYCNAIA AGGAKEWDFAWSEFKNATIASEAEKLRSALSCTKQPWLLNRYLEYTLDPDMIRKQDATSTIVYIANNVVG QSLAWDFVRDQWSFIFSQYGGGSFSFSNLINGVTKRFSSDFELKQLQQFKADNSEVGFGSGTLAVDQSIE RTNANIKWIAENKENVLKWFSVEAML

>XP_028439978.1 aminopeptidase N-like isoform X2 [Perca flavescens]

MKGRGVPPGLLWIATLCALTTVEALGKASVPNISPRQDGSQEEKAKNNNQVLPTDGGPTSKPPNTTPSPS TSPSGPWDNYRLPKSLVPINYNLTLWPRLTPDENGQYIFTGLSIVEFECVEETDLILIHSNKLNYTLKND HLAELTSVNSGVRAPSIKSSWLQSVTQYLVLQLDGKLIKGHRYHLYTDFTGELADDLGGFYRSEYFEDGV KKVVATTQMQPTDARKAFPCFDEPAMKAIFHITLIHDKGTVALSNGEEKESSVIEGQDLLKTAFKPTEKM STYLLAFIVSDYTFINNTVDGVLIRIFARKPAIDAGQGEYALNKTGPILKFFEEYYNSSYPLPKSDQIAL PDFNAGAMENWGLITYRETALLYDKDSSSNSNKERIATIIAHELAHMWFGNLVTLRWWNDLWLNEGFASY VEYLGAAHAEPNWNLTDLIVLSDVHRVFAVDALASSHPLSSKEEDIQKPAQISELFDAISYSKGAAVLRM LSDFLTEDVFTMGLRTYLAEFAFENAVYTDLWKHLQKAVDLSVTKLPDTVEKIMNTWVLQMGFPVVTINT QTGLVSQEHFLLDPDSNVTTESPFNYEWIVPIKWMKTGIIKEPQWLKVKSSTIDAMKASGTEWVLANLNV VGYYRVNYDEGNWNKLLNCLMADHKLIPVINRAQLVDDAFNLARAKIIPTVWALNTTKYLNKETEYMPWK SALNNLDFFYLMFDRSEVYGPMQNYLRKQVTPLFQYYKNMTGNWRDVPDGHMDQYNQVNAISLACRTGLE ECQNLTKTWFNQWMDTKMNPIHPNLRSTVYCNAIAAGDAKEWEFAWDEFQNATIASEAEKLRSALACTNQ PWLLNRYLEYTLMPNLIRKQDATSTIVYIANNVVGQSLAWDFVRARWSYIFKEYGGGSFSFSNLINGVTK RFSTEFELQQLKQFKADNSEIGFGSGTLAVDQSIERTMSNIKWITENKQNVLDWFDN

>XP_028257939.1 aminopeptidase N-like [Parambassis ranga]

MGKVYYVSKNMGLGMLVLAGTALTTIIALSIAYDKEKAKNQSKPGNGVTSSSGTPIPPTTPFTPKEPWDH YRLPDSLTPVSYNVTLWPRLEPNADGLYIFTGHSAVVFKCMKETDLIIIHSNKLNLTTFLGHHAKLSSLG QVTAPAIQRTWLVEKTEFLVVQLKSRLAVGASYVLQTEFLGELADDLEGFYRSEYMEDGVKKVVATSQMQ ATYARKTFPCFDEPAMKATFNVTIIHSRGTLALSNGREIDMSDSDIDGVPVRVTRFEPTERMSTYLLAFI VSEFVSIQSHQNNLMIRIWAKRKAISDRQGEYALNVTGPILQFYEQYYNTAYPLSKSDQIALPDFNAGAM ENWGLVTYRETALLYDPILSSTGNKERVTTVISHELAHMWFGNLVTLRWWNDLWLNEGFASYVEYLGADY AEPTWSIKDQIILYDMQKVFAVDALASSHPLSRHEDEVNSPAQISEMFNTISYSKGAAVLRMLSEFLTES VFARGLSSYLNTFAFGNTVYTDLWDHLQQAVRDTPAIHIPHSVHDIMNRWTLQMGFPVVTIDTRTGTVTQ KHFLLDPDSVVDRPSQFNYTWFVPIKWMKSSVEQQQYWLLQKTDTCSQMRVSGEEWVLANTNASGYFRVN YDLDNWGRILSLLSSNHQYLSIINRAQIIDDAFNLARAKIISTTLALKTTKYLSKERDYIPWESALRNLD YYILMFDRTEVYGALQAYLKKQIQPLFEYFKTLTANWTKVPTGHTDQYNQINAVGIACRVGVEECRELIK SWYRQWMKNPSHNPIHPNLKSTVYCHAIALGGVEEWDFAWSMFKNATLASEASRLRSAMACTKAPWLLNR YLEYTLDPAKIRKQDATSTIQYIARNVVGMPLAWNFVREKWSYIFNQYGRGSFSFSSLVNGITKRFSTEF ELQELKKFKEDNIHVGFGSATLALEQAIEKTTANIKWVTENKAEVLKWFTEEST

>XP_028305329.1 aminopeptidase Ey-like isoform X1 [Gouania willdenowi]

MGKHCGVNRLCFLGTVLALASVATIVTLWTIALTGGGDDVTAPWDSYRLPSALFPLHYNITLWPQLDTST NTYLFTGSSSVLFQCEVETDLILIHVNKLNLTTLDGSHMARLSAAAGDFVPDILSCWLQTNTQYLVVHLS NKLKPGHSYQLYTEFRGELADDLAGFYRSEYIEDGVRKIVAISQMHPTHARKTFPCFDEPAMKAVFHITL LHTPGSVALSNGLEKDVLNITVDGVNVTQTVFEPTEKMSSYLLAFVVSDYTPLQTTHGDTLMRVWARTKA IEQGQGAYALNVSGPILDFLQLYYNISYPLKKADQVAVPDFYFGAMENWGLVMYRETKLLYDPMSSSNGN KETTVAIIAHELAHMWFGNLVTLRWWNEVWLNEGFASYVSYLGADHAEPTWNIKDLIFLNDIQGVLEVDS LSSSHPLSSEEDSIVLPEQILAQFDIISYSKGAAVLRMLSDFLSESVFVEGLRGYLTHFSYSNTVGTDLW EHLQMVVENNSVSLPQSVGVIMNPWVLQEGYPLVTIDTIQGKVSQRRFILNPEEQVTSRLPRRAEWAVPL RWMKDGEAQTEIWWLMEKEDVNLDMRSRASWVLANVNMSGFYRVNYDFGNWERLLTQLNTDHQIIPLINRAQLIDDLFNLARAQVVPTPLALRASTYLQQEKGYIPWRSALNNLQYLYLMLDRTEAYPLLQDYLRKLITP LYIHYNNTTDNWRQTPEQHMDQYNQVNVLHAACRASLSDCVSLTSDWFRRWMDNPENNTILPNLRPAVYC SAVSAGGNAEWEFTLDQFTRSTVSSERSHLLSALSCSSDAGLLHRLLSFTLSSSVLRWQDGATVIASVAE NRHGNNVSWTFIREHWEHIHTHYGSSIISEVTERFSTPDELQQLKDFVEQVGLESSSAVEQVLERTRANI RWLQHNKEPILHWLQQNQETVLH

>NP_001141.2 aminopeptidase N precursor [Homo sapiens]

MAKGFYISKSLGILGILLGVAAVCTIIALSVVYSQEKNKNANSSPVASTTPSASATTNPASATTLDQSKA WNRYRLPNTLKPDSYRVTLRPYLTPNDRGLYVFKGSSTVRFTCKEATDVIIIHSKKLNYTLSQGHRVVLR GVGGSQPPDIDKTELVEPTEYLVVHLKGSLVKDSQYEMDSEFEGELADDLAGFYRSEYMEGNVRKVVATT QMQAADARKSFPCFDEPAMKAEFNITLIHPKDLTALSNMLPKGPSTPLPEDPNWNVTEFHTTPKMSTYLL AFIVSEFDYVEKQASNGVLIRIWARPSAIAAGHGDYALNVTGPILNFFAGHYDTPYPLPKSDQIGLPDFN AGAMENWGLVTYRENSLLFDPLSSSSSNKERVVTVIAHELAHQWFGNLVTIEWWNDLWLNEGFASYVEYL GADYAEPTWNLKDLMVLNDVYRVMAVDALASSHPLSTPASEINTPAQISELFDAISYSKGASVLRMLSSF LSEDVFKQGLASYLHTFAYQNTIYLNLWDHLQEAVNNRSIQLPTTVRDIMNRWTLQMGFPVITVDTSTGT LSQEHFLLDPDSNVTRPSEFNYVWIVPITSIRDGRQQQDYWLIDVRAQNDLFSTSGNEWVLLNLNVTGYY RVNYDEENWRKIQTQLQRDHSAIPVINRAQIINDAFNLASAHKVPVTLALNNTLFLIEERQYMPWEAALS SLSYFKLMFDRSEVYGPMKNYLKKQVTPLFIHFRNNTNNWREIPENLMDQYSEVNAISTACSNGVPECEE MVSGLFKQWMENPNNNPIHPNLRSTVYCNAIAQGGEEEWDFAWEQFRNATLVNEADKLRAALACSKELWI LNRYLSYTLNPDLIRKQDATSTIISITNNVIGQGLVWDFVQSNWKKLFNDYGGGSFSFSNLIQAVTRRFS TEYELQQLEQFKKDNEETGFGSGTRALEQALEKTKANIKWVKENKEVVLQWFTENSK

>NP_032512.2 aminopeptidase N [Mus musculus]

MAKGFYISKTLGILGILLGVAAVCTIIALSVVYAQEKNRNAENSATAPTLPGSTSATTATTTPAVDESKP WNQYRLPKTLIPDSYRVILRPYLTPNNQGLYIFQGNSTVRFTCNQTTDVIIIHSKKLNYTLKGNHRVVLR TLDGTPAPNIDKTELVERTEYLVVHLQGSLVEGRQYEMDSQFQGELADDLAGFYRSEYMEGDVKKVVATT QMQAADARKSFPCFDEPAMKAMFNITLIYPNNLIALSNMLPKESKPYPEDPSCTMTEFHSTPKMSTYLLA YIVSEFKNISSVSANGVQIGIWARPSAIDEGQGDYALNVTGPILNFFAQHYNTSYPLPKSDQIALPDFNA GAMENWGLVTYRESSLVFDSQSSSISNKERVVTVIAHELAHQWFGNLVTVAWWNDLWLNEGFASYVEYLG ADYAEPTWNLKDLMVLNDVYRVMAVDALASSHPLSSPADEIKTPDQIMELFDSITYSKGASVIRMLSSFL TEDLFKKGLSSYLHTYQYSNTVYLDLWEHLQKAVNQQTAVQPPATVRTIMDRWILQMGFPVITVNTNTGE ISQKHFLLDSKSNVTRPSEFNYIWIAPIPFLKSGQEDHYWLDVEKNQSAKFQTSSNEWILLNINVTGYYL VNYDENNWKKLQNQLQTDLSVIPVINRAQIIHDSFNLASAKMIPITLALDNTLFLVKEAEYMPWQAALSS LNYFTLMFDRSEVYGPMKRYLKKQVTPLFFYFQNRTNNWVNRPPTLMEQYNEINAISTACSSGLKECRDL VVELYSQWMKNPNNNTIHPNLRSTVYCNAIAFGGEEEWNFAWEQFRNATLVNEADKLRSALACSKDVWIL NRYLSYTLNPDYIRKQDTTSTIISIASNVAGHPLVWDFVRSNWKKLFENYGGGSFSFANLIQGVTRRFSS EFELQQLEQFKADNSATGFGTGTRALEQALEKTRANIDWVKENKDAVFKWFTENSS

>NP_112274.1 aminopeptidase N precursor [Rattus norvegicus]

MAKGFYISKTLGILGILLGVAAVCTIIALSVVYAQEKNRNAENSAIAPTLPGSTSATTSTTNPAIDESKP WNQYRLPKTLIPDSYQVTLRPYLTPNEQGLYIFKGSSTVRFTCNETTNVIIIHSKKLNYTNKGNHRVALR ALGDTPAPNIDTTELVERTEYLVVHLQGSLVKGHQYEMDSEFQGELADDLAGFYRSEYMEGGNKKVVATT QMQAADARKSFPCFDEPAMKASFNITLIHPNNLTALSNMLPKDSRTLQEDPSWNVTEFHPTPKMSTYLLA YIVSEFKYVEAVSPNRVQIRIWARPSAIDEGHGDYALQVTGPILNFFAQHYNTAYPLEKSDQIALPDFNA GAMENWGLVTYRESALVFDPQSSSISNKERVVTVIAHELAHQWFGNLVTVDWWNDLWLNEGFASYVEFLG ADYAEPTWNLKDLIVLNDVYRVMAVDALASSHPLSSPANEVNTPAQISELFDSITYSKGASVLRMLSSFL TEDLFKKGLSSYLHTFQYSNTIYLDLWEHLQQAVDSQTAIKLPASVSTIMDRWILQMGFPVITVNTSTGE IYQEHFLLDPTSKPTRPSDFNYLWIVPIPYLKNGKEDHYWLETEKNQSAEFQTSSNEWLLLNINVTGYYQ VNYDENNWRKIQNQLQTDLSVIPVINRAQIIHDSFNLASAGKLSITLPLSNTLFLASETEYMPWEAALSS LNYFKLMFDRSEVYGPMKRYLKKQVTPLFAYFKIKTNNWLDRPPTLMEQYNEINAISTACSSGLEECRDL VVGLYSQWMNNSDNNPIHPNLRSTVYCNAIAFGGEEEWNFAWEQFRKATLVNEADKLRSALACSNEVWIL NRYLSYTLNPDYIRKQDATSTIVSIANNVVGQTLVWDFVRSNWKKLFEDYGGGSFSFANLIQGVTRRFSS EFELQQLEQFKEDNSATGFGSGTRALEQALEKTKANIKWVKENKDVVLKWFTENS
