# Supplementary material for: The role of the renin-angiotensin system (RAS) in salinity adaptation in Pacific white shrimp (Litopenaeus vannamei)
Source: Front Endocrinol (Lausanne). 2022 Dec 15;13:1089419. doi: 10.3389/fendo.2022.1089419 (PMC9798321; doi:10.3389/fendo.2022.1089419)
Supplement: Supplementary file 4 [file DataSheet_4.docx]

**The sequences of the amplified products for LV-AT1R gene**

> LV-AT1R (Present study)

GAACTGTCGAAGTCTGATCGATAACATTTTTCACCATGGAAATTACCTTACCCAACGCCTCTATCACTCTGAAGGGGATATTCTTCACACATCTGATTCTTACAGTATGGAGCGGCATGTGTGGATGCCTCACCGACAGTTTTGTGCTCTACAACACTATCTTCCTGATCACCATTATGTGGAGTCTGCACCACCGTGAGACCGAAGAGGCTCCCTTCATGGCCTTTTGTGTTAACGTTCTCTCCATCTTGTTTGACATTGTCAATATGTCCTTAAATTGGCCTGCTGTCCAGACTGGAGGTGCCATGACATTTGGTGCAGCCATGGCAGTCATCAACCTGCTAGTGCGGCCTTTTTCAAGCTACCTTCTCTACCGCATAGTCCAGGACCGAGCAGGGTCATACGGCTCCTTTGGACTGCCTTCTGGATTTGACGGCATCTTTGGTGGACGGCGCAGTCCCTATGAGGACATTGACCAGCCTCCTCAGCAGACGCCTTCTGGAATTGATACACAGAATTCTGCAGCTCCAGGGTCGCCACCAGATAACCTCTTTACCACCTAAATACCCAGTTGGCAGTTGGCAGCAAGAAGC

**The protein sequences that used for bio informatics**

**>Present study angiotensin II receptor [Penaeus vannamei]** MEITLPNASITLKGIFFTHLILTVWSGMCGCLTDSFVLYNTIFLITIMWSLHHRETEEAPFMAFCVNVLSILFDIVNMSLNWPAVQTGGAMTFGAAMAVINLLVRPFSSYLLYRIVQDRAGSYGSFGLPSGFDGIFGGRRSPYEDIDQPPQQTPSGIDTQNSAAPGSPPDNLFTT

>XP_042892138.1 type-1 angiotensin II receptor-associated protein-like isoform X3 [Penaeus japonicus]

MEITLPNASITLKGIFFTHLILTVWSGMCGCLTDSFVLYNTILLITIMWSLHHRESDEAPFMAFCVNVLSIMFDIVNMSLNWPAVQTGGSMTFGAAMAIINLLVRPLSSYLLYRIFQDRAGSYGTFGLPSGFDSIFAGGRRSPYEDIDQPPQQTPSGIDTQNAAAPGSPPENLFTT

>XP_037781559.1 type-1 angiotensin II receptor-associated protein-like isoform X1 [Penaeus monodon]

MEITLPNASITLKGIFFTHLILTVWSGMCGCLTDSFVLYNTILLITIIWSLHHRESEEAPFMAFCVNILSIMFDIVNMSINWPAVQTGGAMTFGAAMAVINLLVRPLSSYLLYRIVQDRAGSYGTFGLPSGFESIFGGRRSPYEDIDQPPQQTPSGIDTQNTAAPGSPPDNLFTT

>XP_047481056.1 type-1 angiotensin II receptor-associated protein-like isoform X2 [Penaeus chinensis]

MEITLPNASITLKGIFFTHLILTVWSGMCGCLTDSFVLYNTILLITIIWSLHHRESEEAPFMAFCVNILSIMFDIVNMSLNWPAVQTGGAMTFGAAMAVINLLMRPLSSFLLYRIVQDRAGSYGTFGLPSGFESIFGGRRSPYEDIDQPPQQTPSGIDTQNTAAPGSPPDNLFTT

>NP_000676.1 type-1 angiotensin II receptor [Homo sapiens]

MILNSSTEDGIKRIQDDCPKAGRHNYIFVMIPTLYSIIFVVGIFGNSLVVIVIYFYMKLKTVASVFLLNLALADLCFLLTLPLWAVYTAMEYRWPFGNYLCKIASASVSFNLYASVFLLTCLSIDRYLAIVHPMKSRLRRTMLVAKVTCIIIWLLAGLASLPAIIHRNVFFIENTNITVCAFHYESQNSTLPIGLGLTKNILGFLFPFLIILTSYTLIWKALKKAYEIQKNKPRNDDIFKIIMAIVLFFFFSWIPHQIFTFLDVLIQLGIIRDCRIADIVDTAMPITICIAYFNNCLNPLFYGFLGKKFKRYFLQLKYIPPKAKSHSNLSTKMSTLSYRPSDNVSSSTK KPAPCFEVE

>NP_112247.2 type-1 angiotensin II receptor A [Rattus norvegicus]

MALNSSAEDGIKRIQDDCPKAGRHSYIFVMIPTLYSIIFVVGIFGNSLVVIVIYFYMKLKTVASVFLLNLALADLCFLLTLPLWAVYTAMEYRWPFGNHLCKIASASVSFNLYASVFLLTCLSIDRYLAIVHPMKSRLRRTMLVAKVTCIIIWLMAGLASLPAVIHRNVYFIENTNITVCAFHYESRNSTLPIGLGLTKNILGFLFPFLIILTSYTLIWKALKKAYEIQKNKPRNDDIFRIIMAIVLFFFFSWVPHQIFTFLDVLIQLGVIHDCKISDIVDTAMPITICIAYFNNCLNPLFYGFLGKKFKKYFQLLKYIPPKAKSHSSLSTKMSTLSYRPSDNMSSSAK KPASCFEVE

>XP_008264394.1 PREDICTED: type-1 angiotensin II receptor isoform X1 [Oryctolagus cuniculus]

MMLNSSTEDGIKRIQDDCPKAGRHNYIFVMIPTLYSIIFVVGIFGNSLVVIVIYFYMKLKTVASVFLLNLALADLCFLLTLPLWAVYTAMEYRWPFGNYLCKIASASVSFNLYASVFLLTCLSIDRYLAIVHPMKSRLRRTMLVAKVTCIIIWLLAGLASLPAIIHRNVFFIENTNITVCAFHYESQNSTLPIGLGLTKNILGFLFPFLIILTSYTLIWKALKKAYEIQKNKPRNDDIFKIIMAIVLFFFFSWVPHQIFTFLDVLIQLGVIHDCRIADIVDTAMPITICIAYFNNCLNPLFYGFLGKKFKKYFQLLKYIPPKAKSHSNLSTKMSTLSYRPSDNVSSSSK KPVPCFEVE

>XP_001110548.1 type-1 angiotensin II receptor isoform X2 [Macaca mulatta]

MILNSSTEDGIKRIQDDCPKAGRHNYIFVMIPTLYSIIFVVGIFGNSLVVIVIYFYMKLKTVASVFLLNLALADLCFLLTLPLWAVYTAMEYRWPFGNYLCKIASASVSFNLYASVFLLTCLSIDRYLAIVHPMKSRLRRTMLVAKVTCIVIWLLAGLASLPAIIHRNVFFIENTNITVCAFHYESQNSTLPIGLGLTKNILGFLFPFLIILTSYTLIWKALKKAYEIQKNKPRNDDIFKIIMAIVLFFFFSWIPHQIFTFLDVLIQLGIIRDCRISDIVDTAMPITICIAYFNNCLNPLFYGFLGKKFKKYFLQLKYIPPKAKSHSNLSTKMSTLSYRPSDNASSSTK KPAPCFEVE

>XP_014022053.1 type-1 angiotensin II receptor-associated protein-like isoform X1 [Salmo salar]

MEIPAINLKAIVLVHWLLTVWGCMAWLPPSYAWGNFSVLAVGVWAIAQRDSIDAVLMFLIGIAVTILTDIIHFGIYYPLPEGLSEMNRDTFRFSAGMAILGLVLKPVSCFIIYQMYRERGGDYNVNFGFPSVTRNRDAYQSIDQQDQTASSANPFNQARDTKPGAANSS

>XP_029631013.1 type-1 angiotensin II receptor-associated protein-like isoform X2 [Salmo trutta]

MEIPAVNLKAIVLMHWLLTIWGCMAWLPPSYAWGNFSVLAVGVWAIAQRDSIDAVLMFLIGIAVTMLTDIIHFGIYYPLAEGLAERNRDTFRFSAGMAILGLLLKPVSCFFVYQMYRERGGDYNVNFGFPSVTRNRDAYQSIDQQDQSTSSANPFNQAEGTKPGPPHSY

>XP_029631013.1 type-1 angiotensin II receptor-associated protein-like isoform X2 [Salmo trutta]

MEIPAVNLKAIVLMHWLLTIWGCMAWLPPSYAWGNFSVLAVGVWAIAQRDSIDAVLMFLIGIAVTMLTDIIHFGIYYPLAEGLAERNRDTFRFSAGMAILGLLLKPVSCFFVYQMYRERGGDYNVNFGFPSVTRNRDAYQSIDQQDQSTSSANPFNQAEGTKPGPPHSY

>XP_041745186.1 type-1 angiotensin II receptor-associated protein-like isoform X1 [Coregonus clupeaformis]

MRELQQLCIKSSETSKVERDHTISAVHWPENMEIPAVNLKAIVLVHWLLTIWGCMAWLPPSYAWGNFSVLAVGVWAIAQRDSIDAVLMFLIGIAVTILTDIIHLGIFYPLAEGLAERNRDTFRFSAGMAILGLLLKPVSCFFIYQMYRERGGDYNVNFAGFPSVTRNRDAYQSIDQQDQSTSSANPFNQAEATKPGAPRSY

>XP_017306447.1 type-1 angiotensin II receptor-associated protein isoform X2 [Ictalurus punctatus] MEIPPINLKAILVVHWLLTVWGCIAWLPSSYICANFSVLAVGVWAMAQRDSVDAVLMFLIGLLLTIVIDIVHFSVFYSVSPAGDLFRFSTGMAIFSLILKPFSCFFVYQMYRERGGEYNINFDLETSVDSRCTTDSADTTFLGRRY

>XP_037566920.1 type-1 angiotensin II receptor-associated protein-like [Dermacentor silvarum]

MISIQNPQLKAIFLVHLLLTVWPLQEAWAPQAYLYYNLIYLLMLAWAIHHKDSEEPVFMALAINLGTILMDIVTLGMHFPHQFTFCAGIAIVNLLFRPVSSVFLLRIFNERGGRYADLGIPNFPGGVGSTGRGQYDDIDQTTTQSVPKTGLDTGSPSHVYSSGGDGLPPYSQP

>XP_037513005.1 type-1 angiotensin II receptor-associated protein-like [Rhipicephalus sanguineus] MISIQNPQLKAIFLVHLLLTVWPLQEAWAPQAYLYYNLIYLLMLAWAIHHKDSEEPVFMALAINLGTILMDIVTLGMHFPHQFTFCAGIAIVNLLFRPVSSVFLLRIFNERGGRYADLGIPNFAGGVGSSGRGQYDDIDQTTTQSVPKTGIDTGSPSHAYSSGGDGLPPYSQP

>XP_035720450.1 type-1 angiotensin II receptor-associated protein-like [Vespa mandarinia]

MPNLSSISSYPLKVIFAIHLVLVTWGIQGCWCPKSAMMYNLLFFICLLWAVHNIESDEPLQFALFINVLSIFFDIVTLSVYYPIGLAYASDKFSAAFMIINLVVRVVTSIYLLRIGQARGGSLATMFTPCPAMGITRQDYEDISHPIPQNSDFVGI

>XP_015838149.1 PREDICTED: type-1 angiotensin II receptor-associated protein isoform X2 [Tribolium castaneum]

MPALPQIRNLNLKVIFLSHFVFIALSSMGFWSTSAYLFYNFFFIIFLVWSLIQPQNEEPLQLAIVVNGVSIFLDILLLVMSYPSDAHSAREKFSAAMCILHLIVRPFSTIVLIKNLEERTGSNGLFGEAPQQSSYEDIDRNAPHTSQAASYDFSTAQQI

>XP_019550332.2 type-1 angiotensin II receptor-associated protein-like [Aedes albopictus]

MDVQSAINTPHIRVKLIALIHTCLIAAALNSYWLPAAYQFYNIIFIITLFWAIHSRQSSDAVQIACFINVIGFLLDLFGIILYFPSKGAILSAVFAIFNLALRPFTLLLLHRELTDRGGSLSLATETTGNNPSNYEDIDQ QHQVFTPTILS
