# Supplementary material for: The role of the renin-angiotensin system (RAS) in salinity adaptation in Pacific white shrimp (Litopenaeus vannamei)
Source: Front Endocrinol (Lausanne). 2022 Dec 15;13:1089419. doi: 10.3389/fendo.2022.1089419 (PMC9798321; doi:10.3389/fendo.2022.1089419)
Supplement: Supplementary file 5 [file DataSheet_5.docx]

**The sequences of the amplified products for LV-RR gene**

> LV-RR (Present study)

CAGTCAAGATGGAAGTTCAGACTGTCAGGTGGCTCTCCCTCTTCTCTCTCCTCTACACGGCTCAGTGTGGGGAGGTGACTGTGGCTCACAGTCCTTCAAATCTGCGCTTCGGATCAGCAGGAGTCCTGCGTGCAACCCACCTTGATGATGTCTTAGCAGCCTCTCTTGGTTATACTCCTGAGTCGGCTCCATGGAAGGGTCTGACCATCACATCCCCCTTCAACCTGCCCATAGCTGCAGTCGTAGTTGAGGTGCATGGGGGTGGAGCTAGTGTGCGGCAGGAGGGATCAACCTACAGCCTGAAGGAAGACACAACTCTCGATGATGTTTTCCAGCGCATGAAGGCTGTCATAGGATACAGAGCTCAGAGGGAGACACTCTTCAAACGCATCACTGTGGATGACAATTTGGATGAAGACAAATACCATGGGTCCACCCTTTCATCCGAGGTACTTAGTCCCAATGAGGAGCCTGATTCAACCTTCTTGCGAGAGATGGATGCTCTTGTGGATGCTGCAGAGAAGGTTGGGGATATAACAACCACAGCCCATGGTGGCCAGGACATCATCTTCCTGCAGGTCAATTCTCTGGCACCACTAGTCAAAACATATGGGCCAGAATCAGCAAAGGTTAAGGAAGCAGAAAAGATCCTGAGAAAACAGCTTGTCCAAGTCACAGACATGATGCGCCACATTTATAATGACAGAGTCTTGGTTGCTACGGCTACAGTGGAGCAGCTAGAGGAACTCTCACGATCTTCACGTTCCATTCTCCAGAGTGAGGATGTGAACTTGGAAGACTACAACCTTGCCACCCAGTACACATCAGACTACCCTGCCATATTCAACATTGTCTTGTGGCTGAGCATCATCTTGCTCCTTGCAGTCTTGGCAACATCTGTTGCCATGGCTACCATGGATCCTGGACGTGATTCCATCATTTATCGCATGACCAACCCACGTATGAAGAAAGACAACTAAAGCTGCATATAGTGTATATCAGTTCTTTTATGACCTATTTTTTCTTCCTCATTAATTTTTTTAATATTTTGAGTTAAAGCACAAACTTTTGGAGAAAAAACAGTATATAAATGAGAAGTTAATTACAGAGATCTGA

**The protein sequences that used for bio informatics**

**>Present study renin receptor-like [Penaeus vannamei]** MEVQTVRWLSLFSLLYTAQCGEVTVAHSPSNLRFGSAGVLRATHLDDVLAASLGYTPESAPWKGLTITSP FNLPIAAVVVEVHGGGASVRQEGSTYSLKEDTTLDDVFQRMKAVIGYRAQRETLFKRITVDDNLDEDKYH GSTLSSEVLSPNEEPDSTFLREMDALVDAAEKVGDITTTAHGGQDIIFLQVNSLAPLVKTYGPESAKVKE AEKILRKQLVQVTDMMRHIYNDRVLVATATVEQLEELSRSSRSILQSEDVNLEDYNLATQYTSDYPAIFN IVLWLSIILLLAVLATSVAMATMDPGRDSIIYRMTNPRMKKDN

>XP_047475464.1 ATPase H(+)-transporting accessory protein 2-like [Penaeus chinensis]

MEVQAVRWLSLFSLLYTAQCGEVTVAHSPSNLRFGSAGVLRATHLDDVLAASLGYTPESAPWNGLTITSP FNLPIAAVVVEVHGGGASVRQEGSTYSLKEDTTLDDVFQRMKAVMGYRAQRETLFKRIVVDDYLGEDTFH GSIPSSEVLSLNDEPDSTFLREMAALVDAAEKAGEMTTTNHGGQDIIFLQVNSLAPLVKTYGLESPKVKE AENILRKQLVQVTDMMRHIYNDRVLVATATVEQLEELSRSSRSILQSEDLNLEDYNLATQYTSDYPAIFN IVLWLSIILLLAVLATSVAMATMDPGRDSIIYRMTNPRMKKDN

>XP_042866097.1 ATPase H(+)-transporting accessory protein 2-like [Penaeus japonicus]

MEVQAVRWLSLFSLLYTAQCGEVTVAFSPPTLRFGSAGALRATHLDDVLAASLGYTPESATWKGLTITSPFSLPMAAVVVEVHGGGASVRQEGSTYSLKEDTSLDDVFHHMKAVIGYRAQRETLFKRIVVDDYLEEDTFHSSIPSSEVLSLNEEPDSTFLREMAALVDAAQKTEELTTTTHGGQDIIFLQVNSLAPLVKTYGLESPKVKEAEKILRNQIVRVTDMMRHTYNDRVLVATATVEQLEELSRSARSILQSDDLNLEDYNLATQYTSDYPAIFNIVLWLSIILLLAVLATSVAMATMDPGRDSIIYRMTNPRMKKDN

>XP_037785280.1 ATPase H(+)-transporting accessory protein 2-like [Penaeus monodon]

MEVQAVRWLSLFSLLYTAQCGEVTVAHSPSNLRFGSAGVLRATHLDDVLAASLGYTPESAPWKGLTITSPFNLPIAAVVVEVHGGGASVRQEGSTYSLKEDTTLDDVFQRMKAVMGYRAQRETLFKRIIVDDYLDEDTFHGSIPSSEVLSLNEEPDSTFLREMAALVDAAEKAGEMTTTNHGGQDIIFLQVNSLAPLVKTYGLESPKVKEAEKILRKQLVQVTDMMRHIYNDRVLVATATVEQLEELSRSSRSILQSEDLNLEDYNLATQYTSDYPAIFNIVLWLSIILLLAVLATSVAMATMDPGRDSIIYRMTNPRMKKDN

>XP_018572333.1 renin receptor [Anoplophora glabripennis]

MLELLISLLFISSAYGAGEFSVLHSPESLTFKGHDHVRESTLKEVYSAALGFSTEHYSNWQGLYIENPFDLPEAIVTVSVDGVADIGQQTGHHYPLRTDEEEVEIYQSLERRISERFPEQNTNLVRIDLSNGLDEVKKYAIFEEIRSENPKHVSHKSLKLDVDEDRQFLKEITLLREITRKIEQGVVKQDHVPDVFWFRVSALHPLSDLHGENSTETTEAKQLLNEAILRLKDAFVKAYDDKALVNVITSDASHTRRTRSILQISAKADEETDKVKYNLASYYSKDYPVIFNIILWFGIAMLFSLIAICLAIGNMDPGRDSIIYRMTSTRMKKDN

>XP_012224211.1 PREDICTED: renin receptor isoform X1 [Linepithema humile]

MCRQLLLLFATLAAVYANDEFVILHSPDSVEFTGNGPIEQSSLKEIMCAALGFTGKQKDTEHSIAVWDPFSMPKALVAIPIDGIEFMPFLKDKTTYPLIVDEVEETTWQAIRSRIEERSNDNTLIRINLSDGVDALGQSALGELKLANMGKLKALNSDVEEDRKFIEEIQLLHAIADKASAKKHECGTDIYWLVVSALKSVMDFHGNTSAAANESYTLLNDAMERVSNAFVNTYDGKVVIVAFTNDASKMRNTRSVLYERQRRDTAYKTNNVKSTGRDKGTPISSDINDNFLVKRDGERDGQNKVKDEEEINSMSSTSESISAYSTTESTTEQTESKINTNTQTIGQPEEFSNRAKIYTENYPVIFNIILWFGVVFFFSLLAICIAIADMDPGRDSIIYRMTSNRMKKDN

>XP_012140295.1 PREDICTED: renin receptor isoform X2 [Megachile rotundata]

MLKLFVCFIAIFIAVAANGDFVVLHSPNSVLFHGNEEVEQSLLKEVLAAALGFTVKLRGVWSGISITDPFSLPEAVVAVVIEGIDSLDIPKGKRFPLNVNEVEETTWQALRGRLEERDNDNTLVRISLGDGLDALGQSALGELKPTPIDESSLRALSLRKEEDKKFLEEVQLLHAIAKKAPSAIKPDSKSDIYWLVISGLRPIFDVYGSNSTASKEALSLLNNALNVIHDAFIQAYDGQVLIVAFTNDASKVHHIRSVTQERQKRETLDTDGKEVANKTNNIESSTDVKHNNNNTNSFNANMLSKQDEHQGDNTKLTNEEDKDTNIDKPEHFDTNGDTNVSNHNDNGDINNNTNLTNGENIVKSNRQSIGQEIKNLAKEYSTDYPAIFNIILWFGVVFVFSLLAICIAIAQMDPGRDSII YRMTSNRMKKDN

>XP_011349190.1 uncharacterized protein LOC105286143 isoform X1 [Ooceraea biroi] MFRQLSLLFTVLAAVHASGEFVVLHSPDSVEFTANGEIEQSNLKEVLSAALGYTGKQKEGEYSIAIMDPF ALPKALVAIAIDGIETLPFLENEAKITYPLIVDEVEETTWQAIRSRVEERSNDNTLVRINLSDGVDALGQ SALGELKLANMEKLKALNSEVEEDHKFVEEMQLLHAIADKASAAKKHDSSTDVYWLVVSALKPVLDLHGN TSETAAEAYTLLNDAMEHVSKAFVNTYDGKAVIAAFTNDASKVRNTRSASLERQRRETPTTAYKTNSIKT TGGAKGAPISSDNNDNLFFKRDGEQDTPSNIKDHKEEIDSMNNTNKSNTDDNSKQPVVQEPSESVNTDKT NTNIQTGQPEFSGRAKTYTENYPVIFNIILWFCIVFFFSLLAICIAIADMDPGRDSIIYRMTSNRMKKDN

>NP_005756.2 renin receptor precursor [Homo sapiens]

MAVFVVLLALVAGVLGNEFSILKSPGSVVFRNGNWPIPGERIPDVAALSMGFSVKEDLSWPGLAVGNLFHRPRATVMVMVKGVNKLALPPGSVISYPLENAVPFSLDSVANSIHSLFSEETPVVLQLAPSEERVYMVGKANSVFEDLSVTLRQLRNRLFQENSVLSSLPLNSLSRNNEVDLLFLSELQVLHDISSLLSRHKHLAKDHSPDLYSLELAGLDEIGKRYGEDSEQFRDASKILVDALQKFADDMYSLYGGNAVVELVTVKSFDTSLIRKTRTILEAKQAKNPASPYNLAYKYNFEYSVVFNMVLWIMIALALAVIITSYNIWNMDPGYDSIIYRMTNQKIRMD

>NP_001007092.1 renin receptor precursor [Rattus norvegicus] MAVLVVLLSSLVSSALANEFSILRSPGSVVFRNGNWPIPGDRIPDVAALSMGFSVKEDLSWPGLAVGNLFHRPRATIMVTVKGVDKLALPTGSVISYPLENAVPFSLDSVANSIHSLFSEETPVVLQLAPSEERVYMVGKANSVFEDLSVTLRQLRNRLFQENSVLNSLPLNSLSRNNEVDLLFLSELQVLHDISSLLSRHKHLAKDHSPDLYSLELAGLDELGKRYGEDSEQFRDASRILVDALQKFADDMYSLYGGNAVVELVTVKSFDTSLVRKSRTILETKQENTQSPYNLAYKYNLEYSVVFNLVLWIMTGLALAVIITSYNIWNMDPGYDSIIYRMTNQKIRMD

>NP_081715.1 renin receptor precursor [Mus musculus]

MAVLVVLLFFLVAGALGNEFSILRSPGSVVFRNGNWPIPGDRIPDVAALSMGFSVKEDLSWPGLAVGNLFHRPRATIMVMVKGVDKLALPAGSVISYPLENAVPFSLDSVANSIHSLFSEETPVVLQLAPSEERVYMVGKANSVFEDLSVTLRQLRNRLFQENSLLNSLPLNSLSRNNEVDLLFLSELQVLHDISSLLSRHKHLAKDHSPDLYSLELAGLDELGKRYGEDSEQFRDASKILVDALQKFADDMYSLYGGNAVVELVTVKSFDTSLVRKSRTILEAKQENTQSPYNLAYKYNLEYSVVFNLVLWIMIGLALAVIITSYNIWNMDPGYDSIIYRMTNQKIRID

>XP_028836891.1 renin receptor-like [Denticeps clupeoides]

MKRPSIYSLLVACTSIFCGVLGDRLTVLRSPSYVTFLDGQWPISGEKVPSLVALTMGFSVHEDLDWPGLQAGPLFQRPRANVLVVVRGVDSLSLPNNITSYPLDNPVPFTLDSVASTVHTLFADSTPVVLQLAPSEERLYMMGMANTVFEDLPVTLQQIRGRLSQEGSILNSLPVNSLSRNNEADLLFLSEIQVLHDISALLQRHRHLAKDHAPDLYSLELSGLEEISRHYGKDSPQFQDATSILASILQKFAADVFGVYSNNAVVEVVTVQNFETPLTRRSRSILATNQISNSQRPYNLAYQYNFQYAVIFNIILWLMIVLVLAVIAIAYNLWNMDPGYDSIIYRMTNQ KIRLD

>XP_029567885.1 renin receptor-like [Salmo trutta]

MLLENRRRMETLFSVAFIFCSVFTAGVQGDSLTILQAPGYVSFQKGDWPISGEKIPDMVALTMGFSVQEDLSWPGLRAGQLFQRPRANVLVVVRGVDSLALPQNVASYPLENPVPFTLDSVAETVHSLFADDTPVVLQLAPSEERLYMLGKANAVFEDLPVTLQQIRSRLSQDGSVLASLPLNSLNRNAEADLLFLSEVQVLHDITALLQRHRHLAQDHSPDLYSLELSGLEELGRRYGQDSPQYRDATAILANVLQKFGEDVFGLYGDSAVVEVVTVKSFEAPLTRKSRSILESKQISNPGSPYNLAYKYNFNYAVIFNIVLWLMIILALAVIVISYNLWNMDPGYDSI IYRMTNQKIRLD

>NP_001133563.1 Renin receptor precursor [Salmo salar]

MSLENRRRMEALFTVALIFCSAFTSGVQGDSLTILQAPEYVSFQKGDWPISGEKIPDMVALTMGFSVQEDLSWPGLRAGPLFQRPRANVLVVVRGVDSLALPQSVASYPLENPVPFTLDSVAETVHTLFAEDTPVVLQLAPSEERLYMLGKANAVFEDLPVTLQQIRARLSQDGSVLTSLPLNSLSRNAEADLLFLSEVQVLHDITALLQRHRHLAKDHSPDLYSLELSGLEELGRRYGQDSSQYQDATAILANVLQKFGEDLFGLYGDSAVVEVVTVKNFEAPLTRKSRSILESKQISNPGSPYNLAYKYNFEYAVIFNIVLWLMIILALAVIVISYNLWNMDPGYDSI IYRMTNQKIRLD

>XP_020333305.1 renin receptor [Oncorhynchus kisutch]

MSLENRRRMEAVFTVALIFCSAFTSGVQGDSLTILQAPEYVSFQKGDWPISGEKIPDMVALTMGFSVQEDLSWPGLRAGPLFQRPRANVLVVVRGVDSLALPQSVASYPLENPVPFTLDSVAETVHTLFAEDTPVVLQLAPSEERLYMLGKANAVFEDLPVTLQQIRARLSQDGSVLASLPLNSLSRNAEADLLFLSEVQVLHDITALLQRHRHLAKDHSPDLYSLELSGLEELGRRYGQDSSQYQDATAILANVLQKFGEDLFGLYGDSAVVEVVTVKNFEVPLTRKSRSILESKQISNPGSPYNLAYKYNFEYAVIFNIVLWLMIILALAVIVISYNLWNMDPGYDSI IYRMTNQKIRLD

>XP_021435593.1 renin receptor [Oncorhynchus mykiss]

MSLENRRRMEAVFTVALIFCSAFTSGVQGDSLTILQAPEYVSFQKGDWPISGEKIPDMVALTMGFSVQEDLSWPGLRAGPLFQRPRANVLVVVRGVDSLALPQSVASYPLENPVPFTLDSVAETVHTLFAEDTPVVLQLAPSEERLYMLGKANAVFEDLPVTLQQIRARLSQDGSVLASLPLNSLSRNAEADLLFLSEVQVLHDITALLQRHRHLAKDHSPDLYSLELSGLEELGRRYGQDSSQYQDATAILANVLQKFGEDLFGLYGDSAVVEVVTVKNFEVPLTRKSRSILESKQISNPGSPYNLAYKYNFEYAVIFNIVLWLMIILALAVIVISYNLWNMDPGYDSI IYRMTNQKIRLD
